# Genetic species identification and population structure of *Halophila* (Hydrocharitaceae) from the Western Pacific to the Eastern Indian Ocean

**DOI:** 10.1186/1471-2148-14-92

**Published:** 2014-04-30

**Authors:** Vy X Nguyen, Matsapume Detcharoen, Piyalap Tuntiprapas, U Soe-Htun, Japar B Sidik, Muta Z Harah, Anchana Prathep, Jutta Papenbrock

**Affiliations:** 1 Institute of Botany, Leibniz University Hannover, Herrenhäuserstr. 2, D-30419 Hannover, Germany; 2 Department of Marine Botan, Institute of Oceanography, Vietnam Academy of Science and Technology, 01 Cau Da, Nha Trang City, Vietnam; 3 Department of Biology, Faculty of Science, Prince of Songkla University, HatYai, Songkhla 90112, Thailand; 4 Seaweed and Seagrass Research Unit, Excellence Centre for Biodiversity of Peninsular Thailand, Faculty of Science, Prince of Songkla University, HaiYai, Songkhla 90112, Thailand; 5 Department of Marine Science, Mawlamyine University, Mawlamyine, Myanmar; 6 Department of Animal Science and Fishery, Faculty of Agriculture and Food Sciences, Universiti Putra Malaysia Bintulu Sarawak Campus, Nyabau Rd, P.O. Box 386, 97008 Bintulu, Sarawak, Malaysia; 7 Department of Aquaculture, Faculty of Agriculture, Universiti Putra Malaysia, 43400 Serdang, Selangor Darul Ehsan, Malaysia

**Keywords:** Eastern Indian Ocean, Evolution, Genetic distance, *Halophila ovalis*, Western Pacific Ocean

## Abstract

**Background:**

The Indo-Pacific region has the largest number of seagrass species worldwide and this region is considered as the origin of the Hydrocharitaceae. *Halophila ovalis* and its closely-related species belonging to the Hydrocharitaceae are well-known as a complex taxonomic challenge mainly due to their high morphological plasticity. The relationship of genetic differentiation and geographic barriers of *H. ovalis* radiation was not much studied in this region. Are there misidentifications between *H. ovalis* and its closely related species? Does any taxonomic uncertainty among different populations of *H. ovalis* persist? Is there any genetic differentiation among populations in the Western Pacific and the Eastern Indian Ocean, which are separated by the Thai-Malay peninsula? Genetic markers can be used to characterize and identify individuals or species and will be used to answer these questions.

**Results:**

Phylogenetic analyses of the nuclear ribosomal internal transcribed spacer region based on materials collected from 17 populations in the Western Pacific and the Eastern Indian Ocean showed that some specimens identified as *H. ovalis* belonged to the *H. major* clade, also supported by morphological data. Evolutionary divergence between the two clades is between 0.033 and 0.038, much higher than the evolutionary divergence among *H. ovalis* populations. Eight haplotypes were found; none of the haplotypes from the Western Pacific is found in India and vice versa. Analysis of genetic diversity based on microsatellite analysis revealed that the genetic diversity in the Western Pacific is higher than in the Eastern Indian Ocean. The unrooted neighbor-joining tree among 14 populations from the Western Pacific and the Eastern Indian Ocean showed six groups. The Mantel test results revealed a significant correlation between genetic and geographic distances among populations. Results from band-based and allele frequency-based approaches from Amplified Fragment Length Polymorphism showed that all samples collected from both sides of the Thai-Malay peninsula were clustered into two clades: Gulf of Thailand and Andaman Sea.

**Conclusions:**

Our study documented the new records of *H. major* for Malaysia and Myanmar. The study also revealed that the Thai-Malay peninsula is a geographic barrier between *H. ovalis* populations in the Western Pacific and the Eastern Indian Ocean.

## Background

The Indo-Pacific Ocean – the origin of seagrass - has the largest number of seagrass species worldwide with huge meadows of mixed species stands, but the taxonomy of *Halophila* members is still unclear and genetic variation has not been much investigated so far [1]. In comparison to other seagrass species in the meadows, *Halophila ovalis* (R. Br.) Hooker is the dominant species and very commonly found in the region. Recently, some new records of *Halophila* members such as *Halophila major* (Zoll.) Miquel, were documented in Southeast Asian countries including Indonesia, Viet Nam and Thailand [2,3]. Additionally, *H. sulawesii* J. Kuo was found and described for the first time in Indonesia [4]. Traditional classification of *H. ovalis* and closely related species based on leaf morphological data is very challenging, and species misidentification among *Halophila* members is reported in various studies [1,5,6]. Genetic markers are considered as helpful tools to resolve boundaries between species as well as the genetic variation among populations within species [6-8].

The Indo-Pacific Ocean also shows a high diversity of landscapes, habitats as well as several existing geographic barriers. Geographic isolation refers to a situation where a species, or a population of a species, becomes separated by a physical barrier, allowing each group to diverge along separate evolutionary paths [9]. The effect of geographic isolation is that the two populations are subjected to different selection pressures, since the conditions in the two areas are different [10]. Thus different alleles will be selected and genetic differences will gradually accumulate between the populations. In general, halophytes such as mangroves, marine algae, and seagrass grow in the coastal zone, which is connective between land and sea [1,11]. Currents along the coast or ocean currents play an important role for the migration of species from one coastal area to another [7]. Recently, there were several studies published on mangroves [12,13] and animals [14,15] from this region revealing the genetic variation isolated by barriers.

Among the members of *Halophila*, *H. ovalis* is widespread in the Indo-Pacific Ocean. In the Pacific, it occurs from southern Japan throughout Southeast Asia, many islands of the western Pacific, and through all but the southern coast of Australia, as well as Lord Howe and Norfolk Islands, and as far east as Tonga and Samoa. In the Indian Ocean, *H. ovalis* is found from southwestern Australia to East Africa and the Red Sea, including Madagascar, with the exception of islands or coastlines with no records. Recently, *H. ovali*s has been also discovered in the Atlantic Ocean on the Island of Antigua [16]. The plant is diminutive and lacks strongly lignified tissue, making it flexible, but vulnerable to physical disturbances [7]. *Halophila ovalis* grows on a variety of substrates and is often the first to colonize newly available sediments [5,17]. The species can grow at a range of temperatures and is distributed from tropical to warm-temperate waters [16,18]. This species has a wide depth distribution as well, with individuals growing from the intertidal up to a depth of 30 m [19]. Like other seagrass species, *H. ovalis* reproduces vegetatively by branching of rhizomes and the formation of new shoots, and sexually through seeds [11]. Due to high variation of leaf morphology and adaptation, Den Hartog [11] emphasized the need for detailed studies of this species to better understand the link between morphological variability and environmental parameters.

Leaf morphology is used as the main key to identify and name *Halophila* species [11,20]. However, traits of leaf morphology are overlapping among members of this genus [1]. Recently, genetic markers of plastid sequences have been used to reveal the genetic relationships among the members of the *Halophila* genus [2,21]. However, the species boundaries could not be fully resolved. Using phylogenetic analyses of the nuclear ribosomal internal transcribed spacer (ITS1-5.8S-ITS2) region showed that some specimens identified as *H. ovalis* belonged to different clades, and this clearly points out the need for critical taxonomic revision of *Halophila* material from the entire geographic distribution of this genus [7]. This nuclear sequence was also used to identify the genetic relation of *H. ovalis* and closely related species namely *H. major, Halophila nipponica* J. Kuo*, Halophila minor* (Zoll.) den Hartog and *Halophila hawaiana* Doty and B. C. Stone [6,7,22,23].

There are several techniques including isozyme analyses [24,25], Random Amplified Polymorphic DNA (RAPD) [26-29], Amplified Fragment Length polymorphism (AFLP) [30-33] and microsatellites [34,35] to access genetic variation among and between seagrass populations. The major advantage of the AFLP technique is the large number of polymorphisms that the method generates compared with other markers. However, the methodology of AFLP experiments and post-run data analysis are complex and time consuming compared with other markers [36,37]*.* Microsatellites are simple sequence repeats (SSRs) with advantages like locus-specificity, co-dominance, high degree of polymorphism, and it is also possible to work with partially degraded DNA [38]. So far there is only little information of DNA fingerprinting techniques applied for *H. ovalis.*

It is hypothesized that (i) taxonomic uncertainty among different populations of *H. ovalis* persists and (ii) geographic distance, differentiation of habitats or the geographic barrier of the Indo-Malay peninsula may affect the genetic variation of *H. ovalis* from the Western Pacific to the Eastern Indian Ocean. The aims of this study are (i) to identify *Halophila* species collected in Hong Kong, Thailand, Malaysia and India based on the molecular marker (ITS1-5.8S-ITS2) and (ii) to search for the genetic structure of *H. ovalis* from the Western Pacific to the Eastern Indian Ocean based on microsatellite and AFLP approaches.

## Results

### Species identification based on the nuclear ITS sequences and morphology

**Figure 1:**
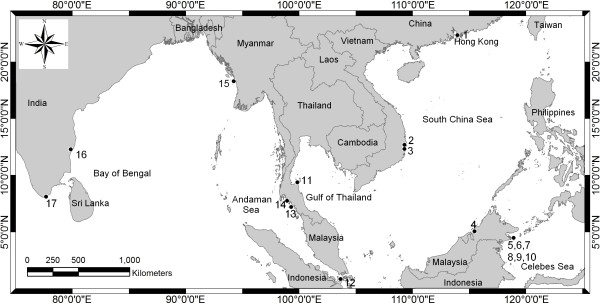
**The map shows the Western Pacific (South China Sea, Celebes Sea and Gulf of Thailand), the Eastern Indian Ocean (Andaman Sea and Bay of Bengal) and the respective countries (Source: The National Oceanic and Atmospheric Administration (*NOAA *), USA, public domain data).** Seventeen sample collection sites are represented as numbers. 1. Tung Chung Bay, Hong Kong (HK-tc), 2. Van Phong Bay, Viet Nam (VN-vp), 3. Thuy Trieu lagoon, Viet Nam (VN-tt), 4. Sarawak, Malaysia (MY-sr), 5. Tiga Island, Malaysia (MY-tg), 6. Mabul Island, Malaysia (MY-mb), 7. Gusungan Island, Malaysia (MY-gs), 8. Sibangat Island, Malaysia (MY-sb), 9. Bodgaya Island, Malaysia (MY-bd), 10. Maiga Island, Malaysia (MY-mg), 11. Kanom, Thailand (TH-kn), 12. Johore, Malaysia (MY-jo), 13. Satun, Thailand (TH-sa), 14. Trang, Thailand (TH-tr), 15. Gyeiktaw, Myanmar (MM-gy), 16. Marakanam, India (IN-ma) and 17. Kanyakumari, India (IN-ka).

**Figure 2:**
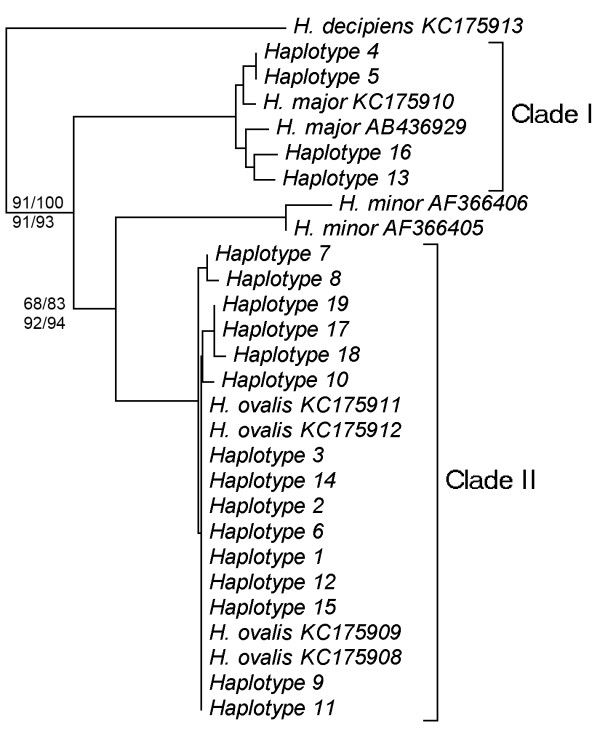
**Phylogeny of members of the *Halophila *genus inferred from Maximum Likelihood, Neighbour Joining, Maximum Parsimony and Bayesian Analysis.** The dataset based on 628 bp (including gaps) of nrDNA sequences comprising ITS-1, 5.8S rDNA, and ITS-2. The bootstrap value of each method is shown in each node: above nodes, left: Maximum Likelihood, right: Neighbor Joining; bellow nodes, left: Maximum Parsimony, right: Bayesian Analysis. See Table 1 for locations of the haplotypes.

Nineteen ITS sequences (Additional file 1) achieved from haplotypes collected at 17 populations of *Halophila* spp. in the study sites shown in Figure 1 and listed in Table 1 were used for the phylogenetic analysis. The alignment of the sequences received from three independent PCRs revealed that there were no nucleotide differences among replications. Fragments of 18S and 28S were removed to gain only the sequence of ITS1-5.8S-ITS2 (620–624 bp). A final alignment of 628 bp (including nucleotides and gaps) was generated for ITS1-5.8S-ITS2, of which 43 (6.8%) were parsimony informative characters, 75 (11.9%) were variable sites, 549 (87.4%) were conserved sites, and 32 (5.1%) were singleton sites. Results of the four algorithms applied (maximum likelihood (ML), neighbor joining (NJ), maximum parsimony (MP) and Bayesian analysis (BA)) showed that all samples collected from the 17 populations were distributed into two clades consisting of *H. major* (clade I) and *H. ovalis* (clade II) with 98, 100, 96 and 99% bootstrap values, respectively. There was no difference in the topology of the phylogenetic trees based on these different methods except for small differences in the bootstrap values. In clade I, haplotypes (Hap.) 4, 5, 13, and 16 clustered with known *H. major* sequences. In clade II, the remaining haplotypes clustered with known sequences from *H. ovalis.* None of the samples clustered with known sequences from *H. minor* (Figure 2). The results also showed that nucleotide differences among individuals of the *H. major* clade and among individuals of the *H. ovalis* clade were zero to six nucleotides and zero to three nucleotides, respectively. However, the counts of different nucleotides between the two clades were 19 to 23. In addition, evolutionary divergence among individuals of the *H. major* clade and among individuals of the *H. ovalis* clade was 0.000 to 0.010 and 0.000 to 0.005, respectively. Evolutionary divergence between the two clades was 0.033 to 0.038. The results clearly indicate that haplotypes 4, 5, 13, and 16 need to be classified as *H. major* and samples collected at TH-tr (Hap. 9) need to be grouped into the *H. ovalis* clade instead with *H. minor* sequences. For both countries, Malaysia and Myanmar, it is the first time that *H. major* was recorded.

**Table 1:** Locations/abbreviations, regions, coordinates, sample size and taxa used in this study

					**Kind of analysis**				
**No.**	**Location**	**Coordinates (degree)**	**Sample size**	**Taxon**	**ITS**	**AFLP**	**SSRs**	**Citations**	**GB number**
1	HK-tc^1^	113.9249°E; 22.2889°N	6	*H. ovalis*	X, Hap.1		X	This study	KF620337^+^
2	VN-vp^1^	109.3445°E; 12.1289°N	10	*H. ovalis*	X		X	[23]	KC175909
3	VN-tt^1^	109.3222°E; 12.1278°N	10	*H. ovalis*	X		X	[23]	KC175908
4	MY-sr^1^	115.4652°E; 04.9825°N	5	*H. ovalis*	X, Hap. 2		X	This study	KF620338^+^
5	MY-tg^1^	118.6006°E; 04.3750°N	5	*H. ovalis*	X, Hap. 3		X	This study	KF620339^+^
6	MY-mb^1^	118.6265°E; 04.2479°N	5	H. major*	X, Hap. 4			This study	KF620340^+^
7	MY-gs^1^	118.5458°E; 04.3161°N	5	H. major*	X, Hap. 5			This study	KF620341^+^
8	MY-sb^1^	118.6626°E; 04.5546°N	5	*H. ovalis*	X, Hap. 6		X	This study	KF620342^+^
9	MY-bd^1^	118.7208°E; 04.6016°N	5	*H. ovalis*	X, Hap. 7		X	This study	KF620343^+^
10	MY-mg^1^	118.6868°E; 04.6080°N	5	*H. ovalis*	X, Hap. 8		X	This study	KF620344^+^
11	TH-kn^1^	099.8802°E; 09.2128°N	4	H. major***	X, Hap. 9	X	X	This study	KF620345^+^
12	MY-jo^1^	103.1333°E; 01.3322°N	5	*H. ovalis*	X, Hap. 10		X	This study	KF620346^+^
13	TH-sa^2^	099.7586°E; 06.7824°N	9	*H. ovalis*	X, Hap. 11	X	X	This study	KF620347^+^
14	TH-tr^2^								
	Site 1	099.3159°E; 07.3745°N	5	*H. ovalis*	X, Hap. 12	X	X	This study	KF620348^+^
	Site 2	099.3159°E; 07.3745°N	6	H. ovalis**	X, Hap. 13	X	X		KF620349^+^
	Site 3	099.3389°E; 07.3829°N	5	*H. ovalis*	X, Hap. 14-15				KF620350-1^+^
15	MM-gy^2^	094.3393°E; 18.3650°N	7	H. major*	X, Hap. 16			This study	KF620352^+^
16	IN-ma^2^	079.9790°E; 12.2330°N	10	*H. ovalis*	X, Hap. 17-18		X	This study	KF620354-5^+^
17	IN-ka^2^	077.5640°E; 08.1001°N	10	*H. ovalis*	X, Hap. 19		X	This study	KF620353
				*H. decipiens*	X			[23]	KC175913
				*H. minor*	X			[7]	AF366405^+^
				*H. minor*	X			[7]	AF366406^+^

There are 122 individuals collected from 17 populations in the Western Pacific and the Eastern Indian Ocean. X: genetic marker used for the populations. *, **, ***: First identification as *H. ovalis, H. minor* and *H. major*, respectively. Hap. 1–19: Haplotypes 1–19. Abbreviations as in Figure 1. ^1^Pacific Ocean, ^2^Indian Ocean. ^+^Accession number for sequences deposited in GenBank.

**Figure 3:**
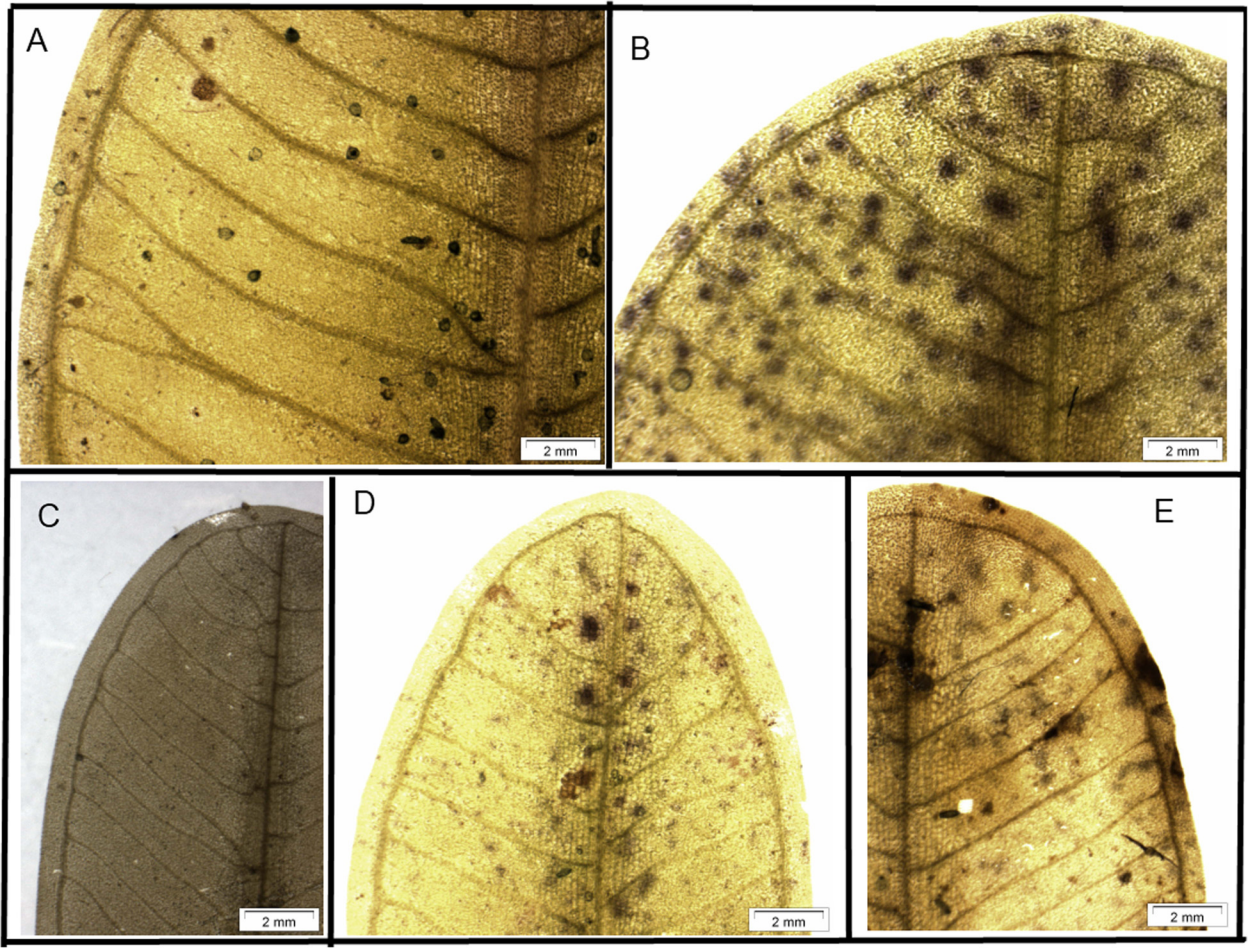
**Leaf morphology of *Halophila ovalis *and *Halophila major *collected at various sites in Malaysia. A** and **B**: *Halophila major* samples collected at MY-gs and MY-mb respectively. **C**, **D** and **E**: *Halophila ovalis* samples collected at MY-bd, MY-tg and MY-sb respectively. The scale bar is 2 mm.

The morphological data also supported the results obtained from the molecular ITS data. For the samples identified as *H. major* based on ITS, five characters of leaf morphology including lamina width, lamina length, number of cross-veins, space between intra-marginal veins, and especially the ratio of the distance between intra-marginal vein (*r*) and lamina margin (*R*) showed clear differences in comparison to *H. ovalis*. The ratio of the distance between the intra-marginal vein and the lamina margin was 1:20.8 to 1:25.6. In contrast, this ratio was 1:12 to 1:16 in *H. ovalis*[4]. Moreover, the number of paired cross veins of *H. major* was 18 to 20 and therefore higher than the number of paired cross veins in *H. ovalis* (14 to 17) [4]. The p-values obtained from Levene’s test of lamina width, lamina length, number of paired cross veins were lower than 0.05 (heteroscedasticity). In contrast, the p-values obtained from Levene’s test of the ratio *r*/*R* was higher than 0.05 (homoscedasticity). Single factor ANOVA shows that for the ratio (*r*/*R*) significant differences can be observed among the collection sites (F = 77.82 > F_crit._, p < 0.001). Details resulting from multiple comparisons of each trait obtained by the Tukey test showed that there were significant differences of the ratio (*r*/*R*) between populations at MY-mb, MY-gs, MM-gt (*H. major*) and the remaining populations (*H. ovalis*). Details of comparisons of the leaf morphology of *H. major* and *H. ovalis* are presented in Table 2 and Figure 3.

**Table 2:** **Comparisons of leaf morphology characteristics of *H. major *** **collected in this study and published data from *H. ovalis***

**Characteristic**	**Species**				
	***H. ovalis***	***H. major *** **MY-mb**	***H. major *** **MY-gs**	***H. major *** **MM-gy**	***H. major***
**Lamina width (mm)**	5-20	12 – 15	12 – 15	13	9-11
**Lamina length (mm)**	10-40 (-70)	18 – 22	18 – 22	22	15 – 25
**No. of cross veins**	10 – 25	18 – 20	18 – 20	20	14 - 17
**Space between intramaginal vein (mm)**	0.1 – 0.3	0.25 – 0.3	0.25 – 0.3	0.3	0.2
**Half lamina width: distance between intramarginal veins and lamina margin ration**	1:12-16	1:21 – 22	1:21 – 22	1:20	1:20-25
**Source**	[5]	This study	This study	This study	[5]

### Genetic diversity and population structure of *H. ovalis* from the Western Pacific to the Eastern Indian Ocean

#### Genetic diversity

Data on observed heterozygosity (H_o_), expected heterozygosity (H_e_) and allelic richness (A) are presented in Table 3. Among populations, the highest expected heterozygosity (H_e_) or genetic diversity in the Western Pacific and the Indian Ocean were found at MY-jo and TH-sa, respectively. The lowest expected genetic diversity in the Western Pacific and Indian Ocean were observed at HK-tc and IN-ma, respectively. Genetic diversity of the populations in the Western Pacific Ocean was slightly higher than of the populations in the Indian Ocean (0.306 vs 0.289). However, there was no significant difference between the oceanic systems (*t*-test, p = 0.78). Likewise, observed heterozygosity and allelic richness in the Western Pacific were slightly higher than in the Indian Ocean (0.552 vs 0.542 and 1.560 vs 1.550, respectively). For the observed heterozygosity and allelic richness, there was no statistically significant difference between both oceanic systems (*t*-test, p = 0.926 and 0.929, respectively).

**Table 3:** **Comparison of genetic diversity among *H. ovalis *** **populations**

**Oceanic system**	**Population**	**Observed heterozygosity (H**_**o**_**)**	**Expected heterozygosity (H**_**e**_**)**	**Allelic richness (A)**
Pacific	HK-tc	0.200	0.109	1.2
	VN-vp	0.800	0.421	1.8
	VN-tt	0.600	0.316	1.6
	MY-sr	0.200	0.111	1.2
	MY-tg	0.600	0.333	1.6
	MY-sb	0.520	0.316	1.6
	MY-bd	0.600	0.333	1.6
	MY-mg	0.600	0.333	1.6
	TH-kn	0.600	0.343	1.6
	MY-jo	0.800	0.444	1.8
	Mean (SE)	0.552 (0.206)	0.306 (0.112)	1.560 (0.207)
Indian	TH-sa	0.600	0.320	1.6
	TH-tr	0.567	0.310	1.6
	IN-ma	0.400	0.211	1.4
	IN-ka	0.600	0.316	1.6
	Mean (SE)	0.542 (0.096)	0.289 (0.053)	1.550 (0.100)

Genetic diversity gained from 14 populations in the Western Pacific (*N* = 10) and the Indian Ocean (*N* = 4). Abbreviations as in Figure 1. Calculation was carried out by the excel microsatellite toolkit [39] and FSTAT [40].

#### Population structure

*Halophila ovalis* populations were markedly differentiated from each other in the Western Pacific and the Eastern Indian Ocean (Table 4). For the Western Pacific region (South China Sea, Celebes Sea and Gulf of Thailand), a significant genetic differentiation among investigated populations was observed.

**Table 4:** **Pairwise comparison of population differentiation among *H. ovalis *** **populations**

	**HK-tc**	**VN-vp**	**VN-tt**	**MY-sr**	**MY-tg**	**MY-sb**	**MY-bd**	**MY-mg**	**TH-kn**	**MY-jo**	**TH-sa**	**TH-tr**	**IN-ma**	**IN-ka**
HK-tc	-	0.912	1.183	2.636	1.100	1.879	1.100	1.100	1.146	0.870	0.794	0.691	2.529	1.183
VN-vp	0.477**	-	0.221	0.858	0.434	0.591	0.434	0.561	0.288	0.508	0.741	0.791	1.375	1.036
VN-tt	0.542**	0.181**	-	1.325	0.547	0.900	0.547	0.858	0.377	0.485	1.046	1.078	1.850	1.375
MY-sr	0.725**	0.462**	0.56**	-	0.800	0.594	0.350	0.350	1.535	0.800	0.953	0.918	2.725	1.725
MY-tg	0.524**	0.303**	0.354**	0.444**	-	0.818	0.200	0.350	0.332	0.157	0.688	0.723	1.525	0.703
MY-sb	0.653**	0.372**	0.474**	0.373**	0.450**	-	0.356	0.356	1.103	0.816	1.169	1.211	2.168	1.502
MY-bd	0.524**	0.303**	0.354**	0.259*	0.167*	0.263**	-	0.050	0.480	0.286	0.688	0.723	1.525	1.014
MY-mg	0.524**	0.360**	0.462**	0.259**	0.259*	0.263**	0.048**	-	0.628	0.414	0.688	0.723	1.525	1.014
TH-kn	0.534**	0.223**	0.274**	0.605**	0.249*	0.524*	0.324**	0.386**	-	0.388	0.976	1.022	1.538	0.686
MY-jo	0.465**	0.337**	0.327**	0.444*	0.136**	0.449**	0.222**	0.293**	0.279**	-	0.464	0.508	1.150	0.765
TH-sa	0.443**	0.425**	0.511**	0.488**	0.408**	0.539**	0.408**	0.408**	0.494**	0.317**	-	0.000	1.296	0.731
TH-tr	0.409**	0.442**	0.519**	0.479**	0.42**	0.548**	0.420**	0.420**	0.506**	0.337**	-0.05^ns^	-	1.280	0.731
IN-ma	0.717**	0.579**	0.649**	0.732**	0.604**	0.684**	0.604**	0.604**	0.606**	0.535**	0.564**	0.561**	-	1.280
IN-ka	0.542**	0.509**	0.579**	0.633**	0.413**	0.600**	0.503**	0.503**	0.407**	0.433**	0.422**	0.422**	0.561**	-

Genetic differentiation F_ST_ (below diagonal) and Slatkin’s genetic distance [41] derived from 14 populations. Statistical significance based on a comparison-wise error rate of α = 0.05 (below diagonal). ns = non-significant, * 0.05 ≥ p > 0.01, ** p < 0.01. Abbreviations as in Figure 1. Data was implemented by Arlequin version 3.5 [42]*.*

For the Western Pacific Ocean, genetic distances among populations in regions I, II, III, IV, V and VI (see Table 1 and Figure 1 for abbreviations) were very high. In detail, the genetic distance between region I and III was the highest (2.636). There were lower genetic distances between region II and region V (0.288 to 0.377). However, the genetic distance between II and VI was lower than the genetic distance between VI and III (0.327 to 0.337 vs 0.444). Within region II, the genetic distance between VN-vp and VN-tt (see Table 1 for abbreviations) was 0.221. In contrast, genetic distances among populations greatly varied from population to population, ranking from 0.05 to 0.818, in which the genetic distance between MY-mg and MY-bd was the lowest and the genetic distance between MY-sb and MY-tg was the highest (Table 4). Results of AMOVA for SSRs variation of *H. ovalis* populations in the Western Pacific Ocean showed significant differentiation among groups (p < 0.01), among populations within groups (p < 0.01) and within populations (p < 0.01) (Table 5). Hence, high genetic distance and statistical differences were not only found among regions, but also among populations in the Western Pacific Ocean. The overall genetic variation from the *Halophila* populations in the Western Pacific Ocean was 0.438 calculated from FSTAT.

**Table 5:** **AMOVA (Analysis of Molecular Variance)**[43]**results for SSR variation at 14 collection sites of *H. ovalis***

**Source of variation**	**d.f.**	**Sum of squares**	**Variance of components**	**Percentage of variation**	**Probability**
Among groups	1	46.1	0.27	17.25	p < 0.01*
Among populations within groups	12	98.4	0.54	34.34	p < 0.01*
Within populations	186	140.9	0.76	48.41	p < 0.01*

Group 1 are the populations from the Western Pacific Ocean and group 2 from the Eastern Indian Ocean. Calculations were conducted in Arlequin 3.5.1.3 [42]. *Significantly different.

For the Eastern Indian Ocean, a very high genetic distance between the two regions VII and IX, ranking from 0.731 to 1.296 was observed (Table 4). For the Andaman Sea, the genetic distance between two populations, TH-sa and TH-tr, was determined as zero and non-significant (p = 0.53). In contrast, the genetic distance between IN-ma and IN-ka was very high and significantly different (1.280, p < 0.001). The results of AMOVA for SSRs variation of *H. ovalis* populations in the two regions VII and IX (see Table 1 for abbreviations) indicated that the percentage of variations among groups, among populations within groups and within populations were 20.85, 28.74 and 50.41%, respectively. Significant difference was just found among groups and among populations within groups (p < 0.01) and there were no significant differences within populations (p = 0.5). Moreover, the results of AMOVA for SSRs variation of *H. ovalis* populations in both oceanic systems (Western Pacific vs Eastern Indian) showed significant differences among groups (p < 0.01), among populations within groups (p < 0.01), and within populations (p < 0.01). The overall genetic variation from the *Halophila* populations in the Eastern Indian Ocean was 0.485 calculated from FSTAT.

**Figure 4:**
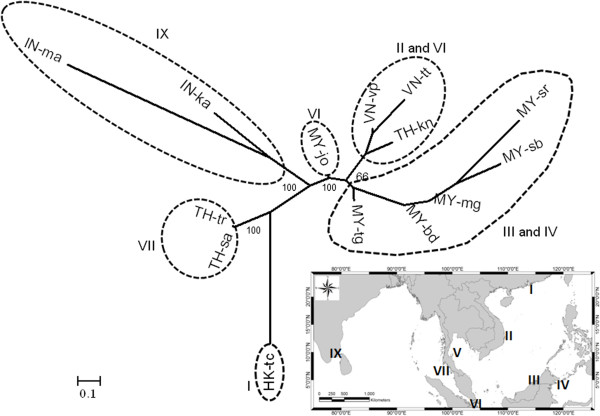
**Unrooted neighbor joining tree illustrating the relationship among *H. ovalis *populations in the Western Pacific and the Indian Ocean.** The tree was based on pair wise Slatkin’s distances [41] and implemented in package Phylip version 3.5 [44]*.* The consensus tree was created using FigTree version 1.3.1 [45]*,* edited and displayed in MEGA5.2 [46]. Abbreviations as in Table 1.

**Figure 5:**
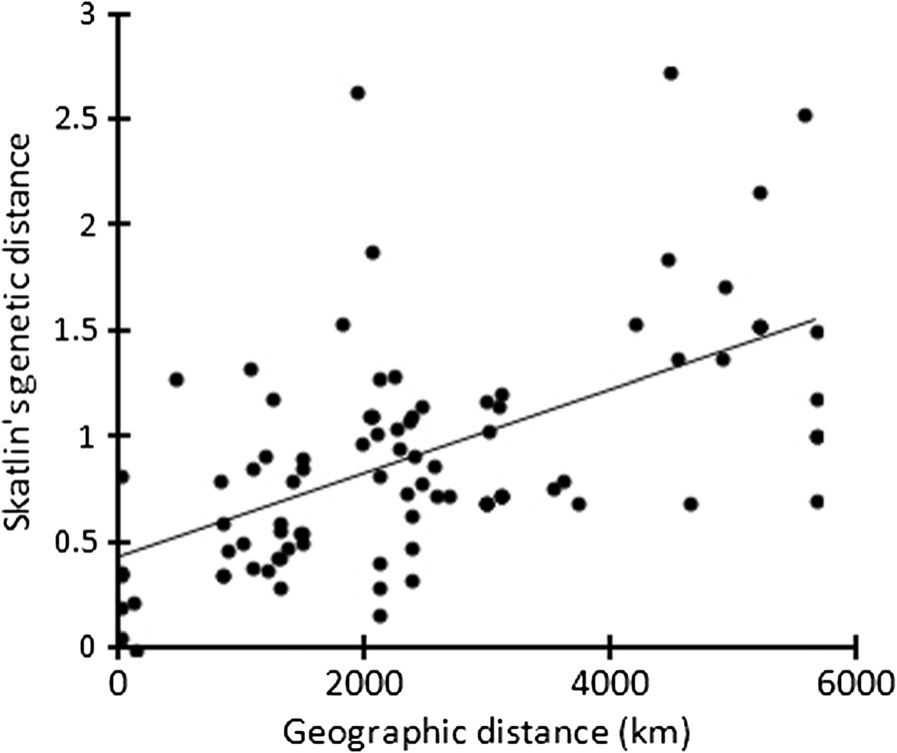
**Correlation between geographic and genetic distance in the study area.** The Mantel test shows significant isolation by distance at significance level of 95%. The Mantel *r* statistic of 0.578 indicates that there is a relatively strong positive correlation between genetic and geographic distance. The p-value < 0.001 indicates that our results are statistically significant at α = 0.05. The chart was implemented by Genepop’007 [47].

The unrooted neighbor-joining tree among 14 populations from eight regions in the Western Pacific and Eastern Indian Ocean showed six main groups including group 1 - Region I: Northern part of the South China Sea (HK), group 2 - Region II and V: Western part of the South China Sea and the Gulf of Thailand (VN-vp, VN-tt and TH-kn), group 3 – Region III and IV: Eastern part of the South China Sea and the Celebes Sea (MY-sr, MY-sb, MY-mg, MY-db and MY-tg), group 4 – Region 6: Southern part of the South China Sea (MY-jo), group 5 – Region VII: Andaman Sea (TH-tr and TH-sa) and group 6 – Region IX: Bay of Bengal (IN-ma and IN-ka) (Figure 4). The multi-locus estimate of spatial differentiation among 14 populations relative to the whole sampled distribution was large (F_ST_ = 0.679). The correlation between geographic and genetic distances in the study area is presented in Figure 5. The result of the Mantel test showed that the geographic distance was linearized and plotted against the geographic distances between populations (r = 0.578, P_Mantel_ < 0.0001, the significance level α = 0.05). An approximately linear increase in Slatkin’s genetic distance with increasing geographic distance between all pairs of populations confirmed a simple model, namely differentiation-by-distance.

**Figure 6:**
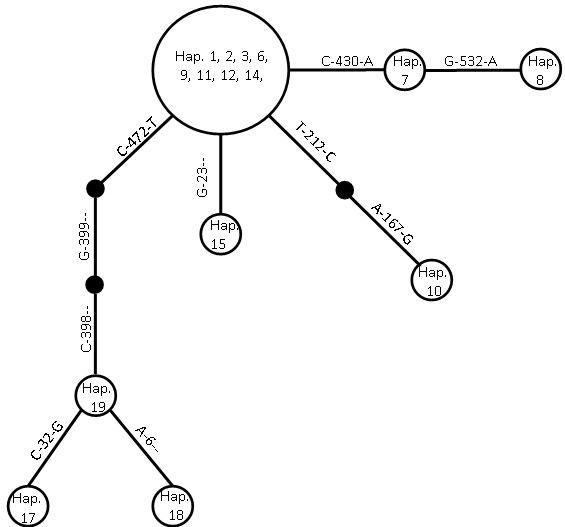
**Haplotype network of eight distinct haplotypes and their distribution detected for *Halophila ovalis *in both Western Pacific and Indian Ocean.** Haplotypes are presented by abbreviations in the circles. Abbreviations as in Table 1. Small solid circles are missing haplotypes. Nucleotide position and differences of nucleotide between two haplotypes are presented in each node. The dendrogram was implemented by software TCS version 1.21 [48].

Based on 15 ITS sequences from *H. ovalis*, there are eight distinct haplotypes found in 14 populations (populations from MM-gy, MY-mb, and MY-gs were rejected because these samples were classified as *H. major*). Haplotype I (including Hap. 1, 2, 3, 6, 9, 11, 12 and 14) commonly occurred in the South China Sea, Celebes Sea and Andaman Sea. Haplotypes II (Hap. 7) and III (Hap. 8) were found in MY-bd and MY-gm (Celebes Sea), respectively, and haplotype IV (Hap. 10) in MY-jo only. In the Andaman Sea, there was one more haplotype present – haplotype V (Hap. 15). Three haplotypes (VI, VII and VIII) that did not occur in the South China Sea, Celebes Sea and Andaman Sea were found in the Bay of Bengal. Haplotype VI (Hap. 19) was identified in IN-ka, while haplotypes VII (Hap. 17) and VIII (Hap. 18) were detected in IN-ma (Figure 6).

For the AFLP analysis, the genetic similarities (Dice index) among the 24 individual *H. ovalis* samples were estimated based on the number of common fragments ranged from 0.560 to 0.928. It also showed that the similarity values of the populations within the Andaman Sea (TH-tr and TH-sa) and within the Gulf of Thailand (TH-kn) were 0.565 to 0.928 and 0.624 to 0.822, respectively. The similarity values between the populations of TH-tr and TH-sa were higher than between the population of TH-tr and TH-kn (0.634 to 0.820 vs 0.582 to 0.731).

**Figure 7:**
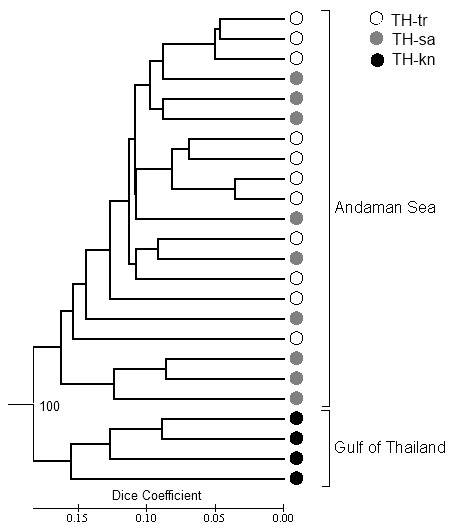
**UPGMA-based dendrogram of *H. ovalis *and closely related species generated from 208 AFLP markers.** The individuals collected in the Andaman Sea and the Gulf of Thailand were divided into two groups with a 100% bootstrap value. There are no significant differences between TR and SA populations. Abbreviations as in Figure 1. The dendrogram was assessed by FreeTree [45] and edited by MEGA5.2 [46].

The cluster analysis (Figure 7) revealed that *H. ovalis* populations were divided into two groups, either collected in the Gulf of Thailand or in the Andaman Sea (100% bootstrap value). However, results of clustering individuals of TH-tr and TH-sa were not significant. The plot of a principal coordinate analysis (PCoA), based on individual genetic distances calculated with 208 AFLP markers, is presented in Additional file 2. The first two axes explained 71.8% and 3.0% of the variation, respectively (explaining 74.8% of total variability). As axis two explained 3% of variance only, it is evident that the remaining axes contribute poorly to explain the variance. Results of PCoA also indicated that *H. ovalis* was clearly distributed in two main clades: Gulf of Thailand clade and Andaman Sea clade.

Based on the above results, the entire samples were organized in two groups: Gulf of Thailand and Andaman Sea group*.* Gene diversity (H) [49] of the entire sample set calculated using POPGENE was 0.272 ± 0.172, while the value of G_ST_[49] was 0.190. Results of AMOVA analysis revealed that genotypic variation was attributable to differentiation between the two groups. The majority of variation among groups was 20.47% (p < 0.01) (Table 6). The matrix of genetic differentiation (F_ST_) among populations of *H. ovalis* revealed that the genetic distance between TH-tr and TH-sa populations (0.137, p < 0.01) was lower than between TH-tr and TH-kn populations (0.335, p < 0.01). The dendrogram based on Nei’s genetic distance also showed that three populations were divided into two main clades: 1) Gulf of Thailand and 2) Andaman Sea (Additional file 3). All data are stored in TreeBASE ( http://purl.org/phylo/treebase/phylows/study/TB2:S15597).

**Table 6:** **AMOVA (Analysis of Molecular Variance)**[43]**results for AFLP variation at three collection sites of *H. ovalis***

**Source of variation**	**d.f.**	**Sum of squares**	**Variance of components**	**Percentage of variation**	**Probability**
Among groups	1	102.9	8.1	20.47	p < 0.01*
Among populations within groups	1	69.7	4.3	10.79	p < 0.01*
Within populations	21	572.8	27.3	68.74	p = 0.3

Group 1 are the populations from the Gulf of Thailand and group 2 from the Andaman Sea. Calculations were conducted in Arlequin 2.2 [42]. *Significantly different.

## Discussion

The present study is the first report of genetic diversity, as well as genetic differences, within and among populations of *H. ovalis* collected from the Western Pacific Ocean to the Eastern Indian Ocean using nuclear sequence comparison (ITS) and two DNA fingerprinting approaches: AFLP and SSRs. Conformation of new records for *H. major* in Malaysia and Myanmar and detection of high levels of polymorphism underlined impressively that genetic markers are powerful tools for species identification and assessing genetic diversity in seagrass.

### New records of *Halophila major* for Malaysia and Myanmar

Variation of leaf morphology has been detected within several species of the *Halophila* genus, namely *H. ovalis*[50,51], *H. hawaiana*[22], and *H. nipponica*[6]. Short et al. [1,52] argued that the taxonomy of *H. major* was unclear, because of overlapping leaf characteristics between *H. ovalis* and *H. major.* Molecular markers, especially ITS, were shown to be a valuable tool in resolving genetic relationships among the species of *Halophila*. For instance, *Halophila euphlebia* Makino was once treated as synonym for *H. ovalis*[11,53]; then, this species was transferred to *H. major*[8]. Results of Uchimura et al. [3] and Shimada et al. [6] supported the conclusion of Kuo et al. [5] that *H. major* and *H. ovalis* are distinct species based on ITS analysis and morphological data. Recently, Short et al. [1] suggested that species in general should be accepted as a new species only if a complete published taxonomic description existed, documenting unique sexual reproductive characters and significant genetic differences. There are three and six species of *Halophila* currently reported in Myanmar and Malaysia, respectively, [17,54] not including *H. major. Halophila major* has been found in the recent years along the coastlines of Southeast Asian countries including Indonesia, Thailand, Viet Nam and Japan [3,23]. As it was demonstrated recently that *Halophila* members could not be fully resolved among closely related species such as *H. ovalis, H. major* and *H. ovata* Gaudich based on concatenated sequences of the two plastid markers *rbc*L and *mat*K [2,21]. In contrast, the phylogenetic analysis of the nuclear ITS sequence indicated that *H. ovalis, H. major* and *H. minor* are distinct species [3,7,23]. Hence, the use of the ITS marker to classify the entire set of samples collected for this study is the best choice based on the current knowledge. In this study, cluster analysis, direct comparison of nucleotide differences and evolutionary divergence between the two clades *H. ovalis* and *H. major* revealed that the materials collected in Mabul Island (MY-mb) and Gusungan Island (MY-gs), both in Malaysia, and one population (MM-gy) in Myanmar differ significantly from the *H. ovalis* clade. Moreover, four methods of constructing phylogenic trees also indicated that materials collected in Mabul Island and Gusungan Island (both in Malaysia) and Myanmar are *H. major*. Only the indications of the molecular methods initiated a detailed microscopic analysis of the leaf samples. The leaf morphology based on the ratio of the distance between the intra-marginal vein and the lamina margin confirmed the ITS analysis. Hence, morphological and nuclear sequence (ITS) analysis indicated that the materials collected in Mabul Island and Gusungan Island (both in Malaysia) and one population in Myanmar are actually *H. major*. In the field this kind of analysis is usually not possible, but our results suggest that careful analysis of seagrass samples need to be conducted before classifying them as *H. ovalis.*

The Indo-Pacific region has the largest number of seagrass species worldwide and this region was considered as the origin of the Hydrocharitaceae family [52,55]. Malaysia not only shows the highest number of *Halophila* species, summing up to seven species [17], this study, but also the highest diversity of *H. ovalis* haplotypes: there are four haplotypes found in six populations in Malaysia. In contrast, Nguyen et al. [23] found only one haplotype in four populations in Viet Nam. This finding is congruent with the hypothesis of Malaysia being the center of origin of the seagrasses.

### Genetic and geographic distance of *H. ovalis* based on SSRs

The genetic diversity indices showed relatively high values from 0.298 to 0.306. Compared to results reported from other studies on seagrass species including *Z. marina* (0.504 to 0.601) [34], (0.310 to 0.460) [56], *Zostera noltii* Hornemann (0.442 – 0.630) [57], *Posidonia oceanica* (L.) Delile (0.191 to 0.363) [58] and *Cymodocea nodosa* (U.) Ascherson (0.286 to 0.564) [59], (0.383 to 0.647) [60] using SSRs markers showed that the genetic diversity of *H. ovalis* is lower. Unfortunately, there are no studies on the genetic diversity of *H. ovalis* based on SSRs markers for direct comparison so far. In other AFLP approaches of seagrass species the genetic diversity such as *Thalassia testudinum* Banks ex König (H = 0.35, [32]) is slightly higher or much lower than in our results, such as for *Z. marina* (H = 0.007 to 0.072, [61]). Hence, the genetic diversity of seagrass varied indeed from species to species, geographic distribution, and different DNA fingerprinting approaches.

The present distribution of the genetic structure within species is influenced by evolutionary history [62]. In this study, genetic and AMOVA analyses indicated significant genetic differences among populations in the Western Pacific Ocean (F_ST_ = 0.483), among populations in the Eastern Indian Ocean (F_ST_ = 0.485), and larger significant differences among 14 populations surveyed in the Western Pacific Ocean and the Eastern Indian Ocean (F_ST_ = 0.679). All above results indicated that great genetic differentiation among populations was detected and/or gene flow among populations is very low. For the Celebes Sea, pair wise genetic differentiation among populations showed genetic differentiation although the geographic distance among populations is about 30 to 40 km. However, genetic differentiation between MY-mg and MY-bd is very low (0.048) in contrast to other populations in the Celebes Sea. This could be explained by the diversity of the habitat such as substratum, currents, and time exposure to air during low tide etc., and those factors may affect the genetic differentiation. Japar et al. [17] stated that there are remarkable variations of *H. ovalis*, which grows in different substratum and depth. Significant genetic differences were also found in *Z. marina* between the Wadden Sea and the Baltic Sea where geographic distance among populations is within areas of 10 to 50 km [34]. Leaf morphology (small form) of *H. ovalis* collected in Tiga Island showed great differences in comparison to the other populations in the Celebes Sea (Prof. Japar, Malaysia, personal observation).

For the South China Sea, there are very great genetic differentiations among populations in the northern part of the South China Sea (HK-tc) and the remaining populations in the western, eastern and southern part of the South China Sea. Perhaps high latitude (or lower average temperature) in the northern part of the South China Sea may lead to the genetic differentiation. Both populations collected in Viet Nam also showed significant differences, although the geographic distance between two populations is less than 100 km. In fact, there are great differences between the environmental conditions from two populations, in the lagoon and in the open sea. It could be explained by the differentiation of salinity, with high salinity (open sea) and low salinity (lagoon). The genetic difference between *H. ovalis* populations in the open sea and the lagoon were also found in India based on AFLP data [33]. For the population of the Gulf of Thailand, the results indicated low genetic differentiation between populations in Thailand and the western part of the South China Sea. Perhaps there was no geographic barrier found between the western part of the South China Sea and the Gulf of Thailand. A study of Morton and Blackmore [63] shows surface currents between the Gulf of Thailand and the western part of the South China Sea, that frequently occur in both summer and winter season.

The genetic differentiation between populations in the Andaman Sea and in the Bay of Bengal is significantly different. This could be explained by a very long geographic distance (more than 2,000 km) between the Bay of Bengal and the Andaman Sea. However, there is no significant genetic differentiation between populations of TH-tr and TH-sa in the Andaman Sea. Perhaps short geographic distances and the same habitat are the main causes that led to the high similarity between the two populations. Results from AFLP analysis also indicated that the genetic distance between populations from TH-tr and TH-sa within the Andaman Sea is much lower than between populations from the Andaman Sea and the Gulf of Thailand. Moreover, surface currents in the winter (from TH-tr to TH-sa) and in the summer (TH-sa to TH-tr) [63] support species dispersal between TH-tr and TH-sa. In contrast, genetic differentiation between populations from IN-ka and IN-ma was also high. It could be explained by the geographic distance as well as habitat differences (lagoon vs open sea).

The result from the unrooted neighbor-joining tree based on Slatkin’s genetic distance showed the identified six main clusters corresponding to populations from different regions. Based on the genetic distance, the population in MY-jo seems to be in between the Western Pacific and the Eastern Indian Ocean, which corresponds to the geographic distribution of *H. ovalis* populations in the study. However, one of the most striking results is the unexpected result in the case of the HK-tc population. It showed no simple relationship between genetic differentiation and distance between pairs of population. The HK-tc population was genetically closer to the population in the Eastern Indian Ocean than to populations in the Western Pacific Ocean. At present we are unable to explain this puzzling result.

### Role of the Thai–Malay Peninsula as a geographic barrier to *H. ovalis* populations in Thailand based on AFLP analysis

Among a total of 231 bands, 208 (90.05%) were polymorphic bands. This contrasts with a level of variability of 30% using AFLP in land plant species, such as rice [64]. In a recent study by Nguyen et al. [33] it was shown that the 17.5% of polymorphic bands are presented in the *H. ovalis – H. ovata* complex. High level of polymorphic bands has previously been reported in *Thalassia testudinum* Banks ex König [32] and *Zostera marina* Linnaeus [61] using AFLP. The percentage of polymorphic bands varies from species to species, geographic distribution, and primer combinations. For the band-based approach performed in this study, the similarity index showed comparable values to the similarity index of *H. ovalis* populations found in India [33]. Comparison between clustering analysis (UPGMA) (Figure 4) and PCoA (Additional file 2) showed that the pattern of clustering the taxa was similar with both analyses: The individuals collected in the Gulf of Thailand clustered as single clade, whereas individuals collected in the Andaman Sea grouped together. AMOVA results (Table 6) also indicate this variation between two groups. In this study, pair wise genetic differentiation (F_ST_) and genetic distance (Additional file 3) among populations support the hypothesis that *H. ovalis* in the Gulf of Thailand and the Andaman Sea are genetically different. The results from AFLP analysis are also in agreement with the results of ITS analysis when different haplotypes in the Gulf of Thailand and the Andaman Sea were classified. In addition, the previous studies on marine animals [12,13] and mangroves [14,15] also indicated that the Thai-Malay peninsula is an effective geographic barrier for populations of different organisms in the Gulf of Thailand and the Andaman Sea.

Based on ITS, AFLP and SSRs analysis of genetic variation of *H. ovalis*, results indicated that the genetic markers are powerful tools to assess the genetic differentiation on the broad sample collection sites. However, the sample size was still low and in the case of TH-kn that may affect the standard error of the diversity in the population of the species as discussed by Singh et al. [65]. According to all our results, missing haplotypes were made visible in the haplotype network, hence we recommend the collection of more samples from populations in the Philippines, somewhere between the two mainlands of Malaysia (Peninsular Malaysia and East Malaysia), and somewhere between the Andaman Sea and the Bay of Bengal (Myanmar and Nicobar Islands) to be included in future studies.

## Conclusion

Our study documented the new records of *H. major* for Malaysia and Myanmar. The study also revealed that the Thai-Malay peninsula is a geographic barrier of *H. ovalis* populations in the Western Pacific and the Eastern Indian Ocean. Characteristics of habitat are also an ecological barrier to the evolution of *H. ovalis* in the smaller scale area.

## Methods

### Sample collection, DNA extraction and morphological analysis

Samplings of *Halophila* species were carried out at the Pacific Ocean and the Indian Ocean. Samples were collected from 17 populations belonging to eight regions depending on the geographic distribution. Regions were determined by long geographic distance (more than 1,000 km in this study) or geographic barrier. Region I (northern part of South China Sea): 1-Hong Kong (HK-tc). Region II (western part of South China Sea): 2-Van Phong (VN-vp), 3-Thuy Trieu (VN-tt). Region III (eastern part of South China Sea): 4-Sarawak (MY-sr). Region IV (Celebes Sea): 5-Tiga Island (MY-tg), 6-Mabul Island (MY-mb), 7-Gusungan Island (MY-gs), 8-Sibangat Island (MY-sb), 9-Bodgaya Island (MY-bd), 10-Maiga Island (MY-mg). Region V (Gulf of Thailand): 11-Kanom (TH-kn). Region VI (southern part of South China Sea): 12-Johore (MY-jo). All above six regions belong to the Pacific Ocean. Region VII (eastern part of Andaman Sea): 13-Satun (TH-sa), 14-Trang (TH-tr). Region VIII (northern part of Andaman Sea): 15-Myanmar (MM-gy). Region IX (Bay of Bengal): 16-Marakanam (IN-ma), 17-Kanyakumari (IN-ka). Details of each sampling site are presented in Figure 1 and Table 1. At each sampling point, plants containing root, rhizome and leaf were selected, and washed with seawater in the field to remove the epiphytes and debris attached to the plants. Each plant sample was placed in a single plastic bag and kept on ice. Plant material was transferred to the laboratory at the same day. In the laboratory, materials were re-washed with de-ionized water to remove seawater. One plant was divided into two parts, one part was pressed as a herbarium voucher specimen and the remaining part was desiccated in silica gel [66] for later DNA extraction. Parts with a length of 10 to 12 cm in a developmentally comparable state from five to ten different plants were haphazardly collected across the beds with a distance of 10 to 15 m among individuals. Materials desiccated in silica gel were brought to the Institute of Botany, Leibniz University Hannover, Germany, for further analysis. Eight to ten young leaves of each individual were homogenized by a bead mill (22 Hz, 2 min), and 100 mg of the fine powdered plant material was used for DNA extraction. DNA extraction was carried out using the Plant Nucleospin II Kit (Macherey & Nagel, Düren, Germany) following manufacture’s instruction with slight modifications according to Lucas et al. [21]. DNA quality was checked on agarose gels stained with ethidium bromide and the concentration was measured by a microplate reader with micro-volume plates (Synergy Mx Multi-Mode, BioTek, Germany).

For the morphological analysis, ten adult leaves collected from ten different individuals from each location were used for the analysis. The five most important and differentiating parameters of leaf morphology including lamina width, lamina length, number of paired cross veins, space between intra-marginal veins and the ratio of the distance between intra-marginal vein (*r*) and lamina margin (*R*) were measured under the microscope Olympus SZ (Olympus, Tokyo, Japan). Photographs were taken using a U-TV1X-2 digital camera (Olympus) connected to a computer. The test for equal variances of each data set of leaf morphology among groups was checked by Levene’s test for homoscedasticity. Levene’s test, one-way analysis of variance (ANOVA), Tukey test was carried out by Minitab software (State College, PA, USA). Specimens were identified using the keys of Kuo et al. [5].

### ITS amplification procedure and sequencing

In this analysis, three individuals per population randomly selected from 15 populations (45 samples in total) described above were used for ITS amplification (Table 1). The region selected for PCR amplification was the nuclear ITS region including the 5.8S sequence. Primer pairs used in this study were (ITS5a) [67] and (ITS4) [68] (Table 7) to amplify a sequence of 700 to 710 bp consisting of ITS1, 5.8S, and ITS2. The total volume of 25 μl included 1x Dream *Taq* Green buffer, 0.2 mM dNTPs, 2 mM MgCl_2_, 1 U *Taq* polymerase (MBI Fermentas, St. Leon-Rot, Germany), 10 to 30 ng template DNA, 1 pmol primer each. The PCR was performed in a PTC 200 thermocycler (Biozym-Diagnostik GmbH, Hess. Oldendorf, Germany) with a heated lid under the following conditions: initial denaturation for 4 min at 95°C followed by 30 cycles of denaturation for 25 s at 95°C, primer annealing for 30 s at 52°C and extension for 35 s at 72°C, terminated by a final hold at 10°C. All PCR reactions were repeated two to four times independently with the same individual to reduce errors, possibly created by the *Taq* polymerase, in the final consensus sequence to a minimum. Direct sequencing of PCR products was done by GATC Biotech (Konstanz, Germany) from both directions. Consensus sequence was achieved by Clone Manager 9 (Sci-Ed, Cary, NC, USA).

**Table 7:** Sequence of primers/adaptors used for ITS, AFLP and SSRs

**Sequence of primers used for ITS**	**Name of primer**	**Ann. temp. (°C)**	**Motive**	**Length of PCR product (bp)**	**Source**
5′-CCTTATCATTTAGAGGAAGGAG-3′	ITS5a	52		700	[67]
5′-TCCTCCGCTTATTGATATGC-3′	ITS4				[68]
Sequence of adaptors and primers used for AFLP					
5′-CTCGTAGACTGCGTACC-3′	*Eco*RI adaptors				[69]
5′-AATTGGTACGCAGTCTAC-3′					
5′-GACGATGAGTCCTGAG-3′	*Mse*I adaptors				
5′-TACTCAGGACTCAT-3′					
5′-GACTGCGTACCAATTCA-3′ (*Eco*RI + A)	Pre-selective primers				
5′-GATGAGTCCTGAGTAAA-3′ (*Mse*I + A)					
*Eco*RI + ACA/*Mse*I + ATC (set1)	Final amplification			50-500	
*Eco*RI + ACC/*Mse*I + ATC (set2)					
*Eco*RI + ACA/*Mse*I + ACA (set3)					
*Eco*RI + ACC/*Mse*I + ACA (set4)					
Sequence of primers used for SSRs					
5′-GAATGGGAAGGTGAAAGAG-3′	HO5	59	(AT)_n_(GA)_n_	260-296	[70]
5′-CACGGCACTGTTCATCTAC-3′						
5′-ATAACCAAAGCCTCCCAAGC-3′	HO8	52	(GA)_n_	156-186		
5′-AAATATCAAACGCCCCTCAC-3′						
5′-CAACTAACCAAACGAGAAAC-3′	HO36	59	(GA)_n_GC(GA)_n_	220-240		
5′-AACCTTGACACCTGCTAATA-3′						
5′-ATCGAACCCAATAGACACCAG-3′	HO48	59	(GA)_n_	196-246		
5′-CAGGCAACTTAGCAAGAAACT-3′						
5′-AGATAAGTTTCACTCCTGTG-3′	HO51	46	(GA)_n_	141-175		
5′-ACCAGAACCAATCAAGAT-3′						

There are four primer pairs used for final amplification in AFLP and five primer pairs used to amplify five loci in SSRs. Ann. temp. Annealing temperature; bp, base pairs.

### SSRs procedure

One hundred individuals (data given from Table 1) collected from 14 populations in the Pacific and the Indian Ocean were used for the analysis. Details of sample size, names of locations and coordinates are presented in Table 1. Among 10 primer pairs suggested by Xu et al. [70], we used five primer pairs resulting in highly polymorphic bands (HO5, HO8, HO36, HO48 and HO51) (Table 7) for PCR. Thirty ng of template DNA was used in each 15 μl PCR including 1x Williams buffer, 0.2 mM dNTPs, 1 U *Taq* polymerase (MBI Fermentas), and 1 pmol primer each. The PCR was performed in a PTC 200 thermocycler (Biozym-Diagnostik GmbH under the following conditions: initial denaturation for 5 min at 94°C followed by 25 cycles of denaturation for 30 s at 94°C, primer annealing for 30 s at 52 to 59°C and extension for 35 s at 72°C, and terminated by a final hold at 10°C. To each sample, 200 μl of dye (98% formamide, 10 mM EDTA, 0.05% pararosaniline) was added. Reactions were heated up to 72°C for 5 min before loading onto 6% AFLP gels (Sequagel XR, National Diagnostics, Hull, England). For running an AFLP gel on the 4300 DNA Analyzer (LI-COR, Biosciences, Germany) manufacture’s instruction were followed. Base pair lengths obtained from visual analysis was resolved with previously published allele lengths [70] and sequencing was performed when necessary.

### AFLP procedure

Samples were collected from three populations from the Andaman Sea and the Gulf of Thailand. Initially, 10 to 15 individuals per population were collected in Thailand for AFLP analysis. Unfortunately, DNA extracted from some plant samples was degraded. Degradation may have been caused by the humid and hot climate during the collection period in Thailand. Meudt et al. [71] indicated that use of degraded DNA could result in poor quality profiles with low reproducibility in AFLP analysis. Hence, only the samples retrieving high quality DNA were subjected for further experiments. According to Pruett and Winker [72], a sample size of 20 to 30 individuals is recommendable for genetic population studies. However, five to six samples are sufficient to obtain a standard error equal to 10% of the diversity in the population of the species [65]. In this study, there are four and twenty samples included from the Gulf of Thailand and the Andaman Sea, respectively, showing high quality of DNA.

Details of sample size, name of locations and coordinates are presented in Table 1. The AFLP procedure was carried out as reported by Vos et al. [69] with few modifications. In brief, genomic DNA (250 ng) was digested with two restriction enzymes in a total volume of 25 μl including 5 U *Eco*RI, 3 U *Mse*I, 1x Restriction Ligation (RL) buffer (10 mM Tris/HCl, 10 mM MgAc, 50 mM KAc, 5 mM DTT, pH 7.5) for overnight at 37°C. Adapters were prepared in a total volume of 5 μl including 50 pmol of *Mse*I adapters, 5 pmol of *Eco*RI adapters, 0.5 mM ATP and 1.2 U of T4 DNA ligase, and 1x RL buffer. The mix of digested DNA and adapters were incubated at 37°C for 3.5 h and then used as a template for PCR. The pre-selective PCR contained 5 μl of template, 1 U of *Taq* polymerase (MBI Fermentas, St. Leon-Rot, Germany), 0.25 mM of each of the four dNTPs, 1x Williams buffer (10 mM Tris/HCl pH 8.3, 50 mM KCl, 2 mM MgCl_2_, 0.001% gelatine) and 50 ng of *Eco*RI and *Mse*I primers with one selective nucleotide (A) in a total volume of 50 μl. The PCR program consisted of twenty cycles of 30 s at 94°C, 30 s at 60°C and 1 min at 72°C, followed by 10 min at 72°C. An aliquot of the reaction mix was diluted 1:20 with 1x TE Buffer (10 mM Tris/HCl pH 7.5, 1 mM EDTA). The selective PCR contained 2.5 μl of the diluted (1:20) product of the pre-selective PCR, 2 mM dNTPs, and 5 U *Taq* polymerase in a total volume of 10 μl. Four primer pairs, *Eco*RI + ACA/*Mse*I + ATC, *Eco*RI + ACC/*Mse*I + ATC, *Eco*RI + ACA/*Mse*I + ACA and *Eco*RI + ACC/*Mse*I + ACA, (Eurofins MWG Operon, Ebersberg, Germany) were used for the selective amplification. The first amplification cycle was carried out for 30 s at 94°C, 30 s at 65°C and 1 min at 72°C. In each of the following 11 cycles, the annealing temperature was reduced by 0.7°C. The last 24 cycles were carried out at an annealing temperature of 56°C, and the final extension step was carried out at 72°C for 10 min. To each sample, 50 μl of dye (see above) was added. Running conditions and instruments were the same as for SSRs.

### Bioinformatic analysis

The obtained ITS sequences and known sequence of *Halophila decipiens* Ostenfeld (KC175913) and *H. minor* (AF366405; AF366406) were aligned by CLUSTAL X [42] and the alignment was further modified by eye. Gaps were considered as missing data. Identical sequences within each species were excluded from the alignment. Additional in-group sequences were obtained from GenBank (Table 1), and included in the alignment. The program jModelTest 0.1.1 [73] was used to find the model of sequence evolution that fitted best with the data set. Phylogenetic analyses were performed using ML, NJ [74] with the model Tamura 3-parameter, MP [75] in MEGA5.2 [46], and BA (Metropolis-coupled Markov chain Monte Carlo method) performed in MrBayes v.3.2 [76]. *Halophila decipiens* was used as out-group, because it is closer to its ancestor than the *Halophila ovalis* complex [7]. In the analyses, trees were tested by the bootstrapping method with 1,000 replications. All phylogenetic trees achieved from analysis were analyzed and exactly constructed by the "tree of trees" approach [77]. Moreover, a network of relationships among haplotypes was constructed as well as a cladogram that showed the nested structure of the haplotypes. This analysis was conducted in software TCS version 1.21 [78]. Only populations determined as *H. ovalis* based on ITS analysis were used for AFLP and SSRs analysis.

For the AFLP analysis, only polymorphic fragments were scored as binary data (1, band present; 0, band absent). The binary scores were manually compared with the pictures to re-confirm presence or absence of bands. A presence/absence binomial matrix of 30 individuals and 201 polymorphic loci was used as basis for the analysis. In this study, the analysis with two approaches including band-based approach (for individual level) and allele frequency-based approach (for population level) [48] was carried out. In the individual level, the similarity among 30 individuals was calculated by the Dice coefficient [79]. A cluster analysis was performed using unweighted pair group method with arithmetic mean (UPGMA) based on the Dice index [79]. Bootstrap values (based on 1,000 re-samplings) were used to estimate the reliability of the clustering pattern. This analysis was carried out in FreeTree software [80]. The dendrogram was edited and displayed by MEGA5.2 [46]. Principal Coordinates Analysis (PCoA) of the correlation matrix was used to further investigate relationships between individuals using NTSYSpc version 2.20 [81]. At the population level, the allelic diversity at each locus was calculated as h = 1 - ∑p_i_^2^, where p_i_ is the frequency of the i^th^ allele [49]. Allelic diversity within each population was the mean allelic diversities among the 114 loci. Nei’s G_ST_[82] was used as a value of genetic differentiation. G_ST_ was calculated using the formula G_ST_ = (H_T_- H_S_)/H_T_[49], where H_T_ represents the total gene diversity and H_S_ represents the gene diversity within populations. Those values and the dendrograms (UPGMA) were assessed by POPGENE 3.2 [83] and MEGA5.2 [46]. In addition, pairwise genetic distances were calculated and used in AMOVA (Analysis of Molecular Variance, [43]). The analyses were conducted with the Arlequin version 3.5 [42].

For the SSRs, genetic diversity was measured for each site using the indices described by Williams and Orth [84]. These indices include: expected heterozygosity under Hardy-Weinberg equilibrium (H_e_) = (Σ expected frequency of heterozygotes at each locus)/(total number of loci); observed heterozygosity (H_o_) = (Σ frequency of heterozygotes at each locus)/(number of individuals); and allele richness (A) = (Σ number of alleles at each locus)/(total number of loci). All those parameters were assessed by Microsatellite Toolkit for Excel [39] and FSTAT version 2.9.3.1 [40]. Deviation from Hardy-Weinberg proportion was tested using a Markov-chain algorithm developed by Guo and Thompson [85] and implemented in the Genepop’007 [47]. Linkage disequilibrium among all pairs of loci for each population and all populations in the Western Pacific and the Indian Ocean was also tested by Genepop’007 [47]. For the population structure, Wright’s F-statistics (F_ST_) was calculated. F_ST_ measures the degree of inbreeding in the subpopulation relative to the total population, and is commonly used to estimate population differentiation. The software FSTAT version 2.9.3.1 [40] was also used for calculation. Significant differences among groups (F_ST_), among populations within groups (F_SC_) and within population (F_CT_) were test by AMOVA (Analysis of Molecular Variance). This analysis was carried out by Arlequin 3.5 [42]*.* Pairwise distances were calculated from allele frequency data using the Slatkin’s distance [41] in Arlequin 3.5 [42]*.* The unrooted neighbor joining tree was constructed using neighbor joining with bootstrap resampling (1,000 replications) in package Phylip version 3.5 [44] and a consensus tree was created using FigTree version 1.3.1 [45]*.* The tree was edited and displayed in MEGA5.2 [46]*.* Geographic distances (km) among populations were determined from NOAA digital map (Figure 1). The genetic-geographic distance matrix was statistically tested for correlation using the Mantel test [86]. This test was carried out by Genepop’007 [47].

## Availability of supporting data

The data sets supporting the results of this article are available in the TreeBASE repository, http://purl.org/phylo/treebase/phylows/study/TB2:S15597.

## Competing interests

The authors declare that they have no competing interests.

## Authors’ contributions

JP, AP and NXV defined the research topic and the experimental design. NXV, MD, PT, USH, MHZ and JSP collected the materials. NXV and MD carried out the laboratory experiments and generated the data. JP and NXV analyzed the data and wrote the manuscript. All authors have contributed to, seen and approved the manuscript.

## Supplementary Material

Additional file 1 ITS sequences (ITS1-5.8S-ITS2) and their Genbank number (KF620337-KF620355). Click here for file

Additional file 2 **Principal Coordinate Analysis (PCoA) based on 208 AFLP markers.** There are two groups including the Gulf of Thailand and the Andaman Sea. Clustering of TH-tr and TH-sa is not significant. Abbreviations as in Figure 1. Symbols as in Figure 4. The matrix plot is processed by NTSYSpc, 2.20 [81]. Click here for file

Additional file 3 **Dendrogram of genetic distances among three populations of *H. ovalis*.** Branch lengths were calculated by Nei [87]. Abbreviations as in Figure 1. Symbols as in Figure 4. Dendrogram was assessed by POPGENE 3.2 [82], edited by MEGA5.2 [46]. Click here for file
