# Supplementary material for: Genetic species identification and population structure of Halophila (Hydrocharitaceae) from the Western Pacific to the Eastern Indian Ocean
Source: BMC Evol Biol. 2014 Apr 30;14:92. doi: 10.1186/1471-2148-14-92 (PMC4026155; doi:10.1186/1471-2148-14-92)
Supplement: Additional file 1 — ITS sequences (ITS1-5.8S-ITS2) and their Genbank number (KF620337-KF620355). [file 1471-2148-14-92-S1.docx]

KF620337

CGTGACGAGCTCGAGTCGGGGCGGGGAATCCAGCATTCTCTCGCCCCGCCTTGCATCCGTGGACCCATCGCTCGCGTTCCTGCGGCGACGGGTCCTACGGATCCTAACGAACCCCCGGCGCAGATCGCGTCAAGGAGTACATGTGCGAACTTGGGCGGGTGCACGGCCGCGCGTCCCCGCGCGGCCCGTGCGGTTCCGCCCCTTCCACGCAGCGAACTGTATGACTCTCGGCAATGGATATCTAGGCTCTCGCATCGATGAAGAACGTAGCGAAATGCGATACTTGGTGTGAATTGCAGAATCCCGTGAACCATCGAGTCTTTGAACGCAAGTTGCGCCCGGAGCCGTCAGGCCGAGGGCACGTCTGCCTGGGCGACCAATAGGCGATCGCTCCCCTCCCCCCACCCCGAATGCGGTGGACTCGGGTGGTGGAAGCGGAAGCTGGCCATCCGTGAGCTTATAGGCATCGCACGATCCTTCGCGGTTGGCTCAATTCTCTCGACGATGTCTTGCCTCTGACGCATCACGTCGCGCGGTGGATCGCACTCGTTGCTTCACGTGCCGTGTCGTCCCGGCGAGACCTCCGTGGCCATTCCCAAAGCTGAGTATCCCCAGGTCAGGC

KF620338

CGTGACGAGCTCGAGTCGGGGCGGGGAATCCAGCATTCTCTCGCCCCGCCTTGCATCCGTGGACCCATCGCTCGCGTTCCTGCGGCGACGGGTCCTACGGATCCTAACGAACCCCCGGCGCAGATCGCGTCAAGGAGTACATGTGCGAACTTGGGCGGGTGCACGGCCGCGCGTCCCCGCGCGGCCCGTGCGGTTCCGCCCCTTCCACGCAGCGAACTGTATGACTCTCGGCAATGGATATCTAGGCTCTCGCATCGATGAAGAACGTAGCGAAATGCGATACTTGGTGTGAATTGCAGAATCCCGTGAACCATCGAGTCTTTGAACGCAAGTTGCGCCCGGAGCCGTCAGGCCGAGGGCACGTCTGCCTGGGCGACCAATAGGCGATCGCTCCCCTCCCCCCACCCCGAATGCGGTGGACTCGGGTGGTGGAAGCGGAAGCTGGCCATCCGTGAGCTTATAGGCATCGCACGATCCTTCGCGGTTGGCTCAATTCTCTCGACGATGTCTTGCCTCTGACGCATCACGTCGCGCGGTGGATCGCACTCGTTGCTTCACGTGCCGTGTCGTCCCGGCGAGACCTCCGTGGCCATTCCCAAAGCTGAGTATCCCCAGGTCAGGC

KF620339

CGTGACGAGCTCGAGTCGGGGCGGGGAATCCAGCATTCTCTCGCCCCGCCTTGCATCCGTGGACCCATCGCTCGCGTTCCTGCGGCGACGGGTCCTACGGATCCTAACGAACCCCCGGCGCAGATCGCGTCAAGGAGTACATGTGCGAACTTGGGCGGGTGCACGGCCGCGCGTCCCCGCGCGGCCCGTGCGGTTCCGCCCCTTCCACGCAGCGAACTGTATGACTCTCGGCAATGGATATCTAGGCTCTCGCATCGATGAAGAACGTAGCGAAATGCGATACTTGGTGTGAATTGCAGAATCCCGTGAACCATCGAGTCTTTGAACGCAAGTTGCGCCCGGAGCCGTCAGGCCGAGGGCACGTCTGCCTGGGCGACCAATAGGCGATCGCTCCCCTCCCCCCACCCCGAATGCGGTGGACTCGGGTGGTGGAAGCGGAAGCTGGCCATCCGTGAGCTTATAGGCATCGCACGATCCTTCGCGGTTGGCTCAATTCTCTCGACGATGTCTTGCCTCTGACGCATCACGTCGCGCGGTGGATCGCACTCGTTGCTTCACGTGCCGTGTCGTCCCGGCGAGACCTCCGTGGCCATTCCCAAAGCTGAGTATCCCCAGGTCAGGC

KF620340

CGTGACGAGCTCGAGTCGGGGCGAGGAATCCAGCATTCCCCCGCCCCGCCTCGCGTCCGCGGATCCATCGCTCGCGTTCCGGCGGCGATGGGTCCTGCGGATCCTAACGAACCCCCGGCGCGGATCGCGTCAAGGAGTACATGTGCGAACTCGGGCGGGCGCACGGCCGCGCGTCCTCGCGCGGCCCGTGCGGTTCCGCCCCTTCCACGCAGCGAACTGTATGACTCTCGGCAATGGATATCTAGGCTCTCGCATCGATGAAGAACGTAGCGAAATGCGATACTTGGTGTGAATTGCAGAATCCCGTGAACCATCGAGTCTTTGAACGCAAGTTGCGCCCGGAGCCGTCAGGCCGAGGGCACGTCTGCCTGGGCGACCAATAGGCGATCGCTCCCCTCCCCCCGCCCCCGACGCGGTGGCCCTGGGTGGTGGAAGCGGAAGCTGGCCATCCGTGAGCTCGTAGGCATCGCAAGATCCTTCGCGGTTGGCTCAATTCTCTCGACGATGTCCTGCCTCTGACGCATCACGTCGCGCGGTGGATCGCACTCGTTGCTTCACGTGCCGTGTCGTCCCGGCGAGACCTCCGTGGCCATTCCCCAAGCTGAGTATCCCCAGGTCAGGC

KF620341

CGTGACGAGCTCGAGTCGGGGCGAGGAATCCAGCATTCCCCCGCCCCGCCTCGCGTCCGCGGATCCATCGCTCGCGTTCCGGCGGCGATGGGTCCTGCGGATCCTAACGAACCCCCGGCGCGGATCGCGTCAAGGAGTACATGTGCGAACTCGGGCGGGCGCACGGCCGCGCGTCCTCGCGCGGCCCGTGCGGTTCCGCCCCTTCCACGCAGCGAACTGTATGACTCTCGGCAATGGATATCTAGGCTCTCGCATCGATGAAGAACGTAGCGAAATGCGATACTTGGTGTGAATTGCAGAATCCCGTGAACCATCGAGTCTTTGAACGCAAGTTGCGCCCGGAGCCGTCAGGCCGAGGGCACGTCTGCCTGGGCGACCAATAGGCGATCGCTCCCCTCCCCCCGCCCCCGACGCGGTGGCCCTGGGTGGTGGAAGCGGAAGCTGGCCATCCGTGAGCTCGTAGGCATCGCAAGATCCTTCGCGGTTGGCTCAATTCTCTCGACGATGTCCTGCCTCTGACGCATCACGTCGCGCGGTGGATCGCACTCGTTGCTTCACGTGCCGTGTCGTCCCGGCGAGACCTCCGTGGCCATTCCCCAAGCTGAGTATCCCCAGGTCAGGC

KF620342

CGTGACGAGCTCGAGTCGGGGCGGGGAATCCAGCATTCTCTCGCCCCGCCTTGCATCCGTGGACCCATCGCTCGCGTTCCTGCGGCGACGGGTCCTACGGATCCTAACGAACCCCCGGCGCAGATCGCGTCAAGGAGTACATGTGCGAACTTGGGCGGGTGCACGGCCGCGCGTCCCCGCGCGGCCCGTGCGGTTCCGCCCCTTCCACGCAGCGAACTGTATGACTCTCGGCAATGGATATCTAGGCTCTCGCATCGATGAAGAACGTAGCGAAATGCGATACTTGGTGTGAATTGCAGAATCCCGTGAACCATCGAGTCTTTGAACGCAAGTTGCGCCCGGAGCCGTCAGGCCGAGGGCACGTCTGCCTGGGCGACCAATAGGCGATCGCTCCCCTCCCCCCACCCCGAATGCGGTGGACTCGGGTGGTGGAAGCGGAAGCTGGCCATCCGTGAGCTTATAGGCATCGCACGATCCTTCGCGGTTGGCTCAATTCTCTCGACGATGTCTTGCCTCTGACGCATCACGTCGCGCGGTGGATCGCACTCGTTGCTTCACGTGCCGTGTCGTCCCGGCGAGACCTCCGTGGCCATTCCCAAAGCTGAGTATCCCCAGGTCAGGC

KF620343

CGTGACGAGCTCGAGTCGGGGCGGGGAATCCAGCATTCTCTCGCCCCGCCTTGCATCCGTGGACCCATCGCTCGCGTTCCTGCGGCGACGGGTCCTACGGATCCTAACGAACCCCCGGCGCAGATCGCGTCAAGGAGTACATGTGCGAACTTGGGCGGGTGCACGTCCGCGCGTCCCCGCGCGGCCCGTGCGGTTCCGCCCCTTCCACGCAGCGAACTGTATGACTCTCGGCAATGGATATCTAGGCTCTCGCATCGATGAAGAACGTAGCGAAATGCGATACTTGGTGTGAATTGCAGAATCCCGTGAACCATCGAGTCTTTGAACGCAAGTTGCGCCCGGAGCCGTCAGGCCGAGGGCACGTCTGCCTGGGCGACCAATAGGCGATCGCTCCCCTCCCCCCACCCCGAATGCGGTGGACTCGGGTGGTGGAAGCGGAAGCTGGCCATCCGTGAGCTTATAGGCATCGCACGATCCTTCGCGGTTGGCTCAATTCTCTCGACGATGTCTTGCCTCTGACGCATCACGTCGCGCGGTGGATCGCACTCGTTGCTTCACGTGCCGTGTCGTCCCGGCGAGACCTCCGTGGCCATTCCCAAAGCTGAGTATCCCCAGGTCAGGC

KF620344

CGTGACGAGCTCGAGTCGGGGCGGGGAATCCAGCATTCTCTCGCCCCGCCTTGCATCCGTGGATCCATCGCTCGCGTTCCTGCGGCGACGGGTCCTACGGATCCTAACGAACCCCCGGCGCAGATCGCGTCAAGGAGTACATGTGCGAACTTGGGCGGGTGCACGTCCGCGCGTCCCCGCGCGGCCCGTGCGGTTCCGCCCCTTCCACGCAGCGAACTGTATGACTCTCGGCAATGGATATCTAGGCTCTCGCATCGATGAAGAACGTAGCGAAATGCGATACTTGGTGTGAATTGCAGAATCCCGTGAACCATCGAGTCTTTGAACGCAAGTTGCGCCCGGAGCCGTCAGGCCGAGGGCACGTCTGCCTGGGCGACCAATAGGCGATCGCTCCCCTCCCCCCACCCCGAATGCGGTGGACTCGGGTGGTGGAAGCGGAAGCTGGCCATCCGTGAGCTTATAGGCATCGCACGATCCTTCGCGGTTGGCTCAATTCTCTCGACGATGTCTTGCCTCTGACGCATCACGTCGCGCGGTGGATCGCACTCGTTGCTTCACGTGCCGTGTCGTCCCGGCGAGACCTCCGTGGCCATTCCCAAAGCTGAGTATCCCCAGGTCAGGC

KF620345

CGTGACGAGCTCGAGTCGGGGCGGGGAATCCAGCATTCTCTCGCCCCGCCTTGCATCCGTGGACCCATCGCTCGCGTTCCTGCGGCGACGGGTCCTACGGATCCTAACGAACCCCCGGCGCAGATCGCGTCAAGGAGTACATGTGCGAACTTGGGCGGGTGCACGGCCGCGCGTCCCCGCGCGGCCCGTGCGGTTCCGCCCCTTCCACGCAGCGAACTGTATGACTCTCGGCAATGGATATCTAGGCTCTCGCATCGATGAAGAACGTAGCGAAATGCGATACTTGGTGTGAATTGCAGAATCCCGTGAACCATCGAGTCTTTGAACGCAAGTTGCGCCCGGAGCCGTCAGGCCGAGGGCACGTCTGCCTGGGCGACCAATAGGCGATCGCTCCCCTCCCCCCACCCCGAATGCGGTGGACTCGGGTGGTGGAAGCGGAAGCTGGCCATCCGTGAGCTTATAGGCATCGCACGATCCTTCGCGGTTGGCTCAATTCTCTCGACGATGTCTTGCCTCTGACGCATCACGTCGCGCGGTGGATCGCACTCGTTGCTTCACGTGCCGTGTCGTCCCGGCGAGACCTCCGTGGCCATTCCCAAAGCTGAGTATCCCCAGGTCAGGC

KF620346

CGTGACGAGCTCGAGTCGGGGCGGGGAATCCAGCATTCTCTCGCCCCGCCTTGCATCCGTGGACCCATCGCTCGCGTTCCTGCGGCGACGGGTCCTACGGATCCTAACGAACCCCCGGCGCAGATCGCGTCAAGGAGTACATGTGCGAACTTGGGCGGGTGCACGGCCGCGCGTCCCCGCGCGGCCCGTGCGGTTCCGCCCCTTCCACGCAGCGAACTGTATGACTCTCGGCAATGGATATCTAGGCTCTCGCATCGATGAAGAACGTAGCGAAATGCGATACTTGGTGTGAATTGCAGAATCCCGTGAACCATCGAGTCTTTGAACGCAAGTTGCGCCCGGAGCCGTCAGGCCGAGGGCACGTCTGCCTGGGCGACCAATGGGCGATCGCTCCCCTCCCCCCACCCCGAATGCGGTGGACTCGGGTGGTGGAAGCGGAAGCTGGCCATCCGTGAGCTTATAGGCATCGCACGATCCTTCGCGGTTGGCTCAATTCTCTCGACGATGTCTTGCCTCTGACGCATCACGTCGCGCGGTGGATCGCACTCGTTGCTTCACGTGCCGTGTCGTCCCGGCGAGACCTCCGTGGCCATTCCCAAAGCTGAGTATCCCCAGGTCAGGC

KF620347

CGTGACGAGCTCGAGTCGGGGCGGGGAATCCAGCATTCTCTCGCCCCGCCTTGCATCCGTGGACCCATCGCTCGCGTTCCTGCGGCGACGGGTCCTACGGATCCTAACGAACCCCCGGCGCAGATCGCGTCAAGGAGTACATGTGCGAACTTGGGCGGGTGCACGGCCGCGCGTCCCCGCGCGGCCCGTGCGGTTCCGCCCCTTCCACGCAGCGAACTGTATGACTCTCGGCAATGGATATCTAGGCTCTCGCATCGATGAAGAACGTAGCGAAATGCGATACTTGGTGTGAATTGCAGAATCCCGTGAACCATCGAGTCTTTGAACGCAAGTTGCGCCCGGAGCCGTCAGGCCGAGGGCACGTCTGCCTGGGCGACCAATAGGCGATCGCTCCCCTCCCCCCACCCCGAATGCGGTGGACTCGGGTGGTGGAAGCGGAAGCTGGCCATCCGTGAGCTTATAGGCATCGCACGATCCTTCGCGGTTGGCTCAATTCTCTCGACGATGTCTTGCCTCTGACGCATCACGTCGCGCGGTGGATCGCACTCGTTGCTTCACGTGCCGTGTCGTCCCGGCGAGACCTCCGTGGCCATTCCCAAAGCTGAGTATCCCCAGGTCAGGC

KF620348

CGTGACGAGCTCGAGTCGGGGCGAGGAATCCAGCATTCCCCCGCCCCGCCTCGCATCCGCGGATCCATCGCTCGCGTTCCGGCGGCGATGGGTCCTGCGGATCCCAACGAACCCCCGGCGCGGATCGCGTCAAGGAGTACATGTGCGAACTCGGGCGGGCGCACGGCCGCGCGTCCTCGCGTGGCTCGTGCGGTTCCGCCCCTTCCACGCAGCGAACTGTATGACTCTCGGCAATGGATATCTAGGCTCTCGCATCGATGAAGAACGTAGCGAAATGCGATACTTGGTGTGAATTGCAGAATCCCGTGAACCATCGAGTCTTTGAACGCAAGTTGCGCCCGGAGCCGTCAGGCCGAGGGCACGTCTGCCTGGGCGACCAATAGGCGATCGCTCCCCTCCCCCCGCCCCCGACGCGGTGGCCCTGGGTGGTGGAAGCGGAAGCTGGCCATCCGTGAGCTCGTAGGCATCGCAAGATCCTTCGCGGTTGGCTCAATTCTCTCGACGATGTCCTGCCTCTGACGCATCACGTCGCGCGGTGGATCGCACTCGTTGCTTCACGTGCCGTGTCGTCCCGGCGAGACCTCCGTGGCCATTCCCCAAGCTGAGTATCCCCAGGTCAGGC

KF620349

CGTGACGAGCTCGAGTCGGGGCGGGGAATCCAGCATTCTCTCGCCCCGCCTTGCATCCGTGGACCCATCGCTCGCGTTCCTGCGGCGACGGGTCCTACGGATCCTAACGAACCCCCGGCGCAGATCGCGTCAAGGAGTACATGTGCGAACTTGGGCGGGTGCACGGCCGCGCGTCCCCGCGCGGCCCGTGCGGTTCCGCCCCTTCCACGCAGCGAACTGTATGACTCTCGGCAATGGATATCTAGGCTCTCGCATCGATGAAGAACGTAGCGAAATGCGATACTTGGTGTGAATTGCAGAATCCCGTGAACCATCGAGTCTTTGAACGCAAGTTGCGCCCGGAGCCGTCAGGCCGAGGGCACGTCTGCCTGGGCGACCAATAGGCGATCGCTCCCCTCCCCCCACCCCGAATGCGGTGGACTCGGGTGGTGGAAGCGGAAGCTGGCCATCCGTGAGCTTATAGGCATCGCACGATCCTTCGCGGTTGGCTCAATTCTCTCGACGATGTCTTGCCTCTGACGCATCACGTCGCGCGGTGGATCGCACTCGTTGCTTCACGTGCCGTGTCGTCCCGGCGAGACCTCCGTGGCCATTCCCAAAGCTGAGTATCCCCAGGTCAGGC

KF620350

CGTGACGAGCTCGAGTCGGGGCGGGGAATCCAGCATTCTCTCGCCCCGCCTTGCATCCGTGGACCCATCGCTCGCGTTCCTGCGGCGACGGGTCCTACGGATCCTAACGAACCCCCGGCGCAGATCGCGTCAAGGAGTACATGTGCGAACTTGGGCGGGTGCACGGCCGCGCGTCCCCGCGCGGCCCGTGCGGTTCCGCCCCTTCCACGCAGCGAACTGTATGACTCTCGGCAATGGATATCTAGGCTCTCGCATCGATGAAGAACGTAGCGAAATGCGATACTTGGTGTGAATTGCAGAATCCCGTGAACCATCGAGTCTTTGAACGCAAGTTGCGCCCGGAGCCGTCAGGCCGAGGGCACGTCTGCCTGGGCGACCAATAGGCGATCGCTCCCCTCCCCCCACCCCGAATGCGGTGGACTCGGGTGGTGGAAGCGGAAGCTGGCCATCCGTGAGCTTATAGGCATCGCACGATCCTTCGCGGTTGGCTCAATTCTCTCGACGATGTCTTGCCTCTGACGCATCACGTCGCGCGGTGGATCGCACTCGTTGCTTCACGTGCCGTGTCGTCCCCGGCGAGACCTCCGTGGCCATTCCCAAAGCTGAGTATCCCCAGGTCAGGC

KF620351

CGTGACGAGCTCGAGTCGGGGCGGGGAATCCAGCATTCTCTCGCCCCGCCTTGCATCCGTGGACCCATCGCTCGCGTTCCTGCGGCGACGGGTCCTACGGATCCTAACGAACCCCCGGCGCAGATCGCGTCAAGGAGTACATGTGCGAACTTGGGCGGGTGCACGGCCGCGCGTCCCCGCGCGGCCCGTGCGGTTCCGCCCCTTCCACGCAGCGAACTGTATGACTCTCGGCAATGGATATCTAGGCTCTCGCATCGATGAAGAACGTAGCGAAATGCGATACTTGGTGTGAATTGCAGAATCCCGTGAACCATCGAGTCTTTGAACGCAAGTTGCGCCCGGAGCCGTCAGGCCGAGGGCACGTCTGCCTGGGCGACCAATAGGCGATCGCTCCCCTCCCCCCACCCCGAATGCGGTGGACTCGGGTGGTGGAAGCGGAAGCTGGCCATCCGTGAGCTTATAGGCATCGCACGATCCTTCGCGGTTGGCTCAATTCTCTCGACGATGTCTTGCCTCTGACGCATCACGTCGCGCGGTGGATCGCACTCGTTGCTTCACGTGCCGTGTCGTCCCGGCGAGACCTCCGTGGCCATTCCCAAAGCTGAGTATCCCCAGGTCAGGC

KF620352

CGTGACGAGCTCGAGTCGGGGCGAGGAATCCAGCATTCCCCCGCCCCGCCTCGCATCCGCGGATCCATCGCTCGCGTTCCGGCGGCGATGGGTCCTGCGGATCCCAACGAACCCCCGGCGCGGATCGCGTCAAGGAGTACATGTGCGAACTCGGGCGGGCGCACGGCCGCGCGTCCTCGCGTGGCTCGTGCGGTTCCGCCCCTTCCACGCAGCGAACTGTATGACTCTCGGCAATGGATATCTAGGCTCTCGCATCGATGAAGAACGTAGCGAAATGCGATACTTGGTGTGAATTGCAGAATCCCGTGAACCATCGAGTCTTTGAACGCAAGTTGCGCCCGGAGCCGTCAGGCCGAGGGCACGTCTGCCTGGGCGACCAATAGGCGATCGCTCCCCTCCCCCCGCCCCCGACGCGGTGGCCCTGGGTGGTGGAAGCGGAAGCTGGCCATCCGTGAGCTCGTAGGCATCGCACGATCCTTCGCGGTTGGCTCAATTCTCTCGACGATGTCTTGCCTCTGACGCATCACGTCGCGCGGTGGATCGCACTCGTTGCTTCACGTGCCGTGTCGTCCCGGCGAGACCTCCGTGGCCATTCCCCAAGCTGAGTATCCCCAGGTCAGGC

KF620353

CGTGACGAGCTCGAGTCGGGGCGGGGAATCCAGCATTCTCTCGCCCCGCCTTGCATCCGTGGACCCATCGCTCGCGTTCCTGCGGCGACGGGTCCTACGGATCCTAACGAACCCCCGGCGCAGGTCGCGTCAAGGAGTACATGTGCGAACTTGGGCGGGTGCACGGCCGCGCGTCCCCGCGCGGCCCGTGCGGTTCCGCGCCCCTTCCACGCAGCGAACTGTATGACTCTCGGCAATGGATATCTAGGCTCTCGCATCGATGAAGAACGTAGCGAAATGCGATACTTGGTGTGAATTGCAGAATCCCGTGAACCATCGAGTCTTTGAACGCAAGTTGCGCCCGGAGCCGTCAGGCCGAGGGCACGTCTGCCTGGGCGACCAATAGGCGATCGCTCCCCTCCCCCCACCCCGAATGCGGTGGACTCGGGTGGTGGAAGCGGAAGCTGGCCATCCGTGAGCTTATAGGCATCGCACGATCCTTCGCGGTTGGCTCAATTCTCTCGACGATGTCTTGCCTCTGACGCATCACGTCGCGCGGTGGATCGCACTCGTTGCTTCACGTGGCGTGTCGTCCCGGCGAGACCTCCGTGGCCATTCCCAAAGCTGAGTATCCCCAGGTCAGGC

KF620354

CGTGACGAGCTCGAGTCGGGGCGGGGAATCCAGCATTCTCTCGCCCCGCCTTGCATCCGTGGACCCATCGCTCGCGTTCCTGCGGCGACGGGTCCTACGGATCCTAACGAACCCCCGGCGCAGGTCGCGTCAAGGAGTACATGTGCGAACTTGGGCGGGTGCACGGCCGCGCGTCCCCGCGCGGCCCGTGCGGTTCCGCGCCCCTTCCACGCAGCGAACTGTATGACTCTCGGCAATGGATATCTAGGCTCTCGCATCGATGAAGAACGTAGCGAAATGCGATACTTGGTGTGAATTGCAGAATCCCGTGAACCATCGAGTCTTTGAACGCAAGTTGCGCCCGGAGCCGTCAGGCCGAGGGCACGTCTGCCTGGGCGACCAATAGGCGATCGCTCCCCTCCCCCCACCCCGAATGCGGTGGACTCGGGTGGTGGAAGCGGAAGCTGGCCATCCGTGAGCTTATAGGCATCGCACGATCCTTCGCGGTTGGCTCAATTCTCTCGACGATGTCTTGCCTCTGACGCATCACGTCGCGCGGTGGATCGCACTCGTTGCTTCACGTGCCGTGTCGTCCCGGCGAGACCTCCGTGGCCATTCCCAAAGCTGAGTATCCCCAGGTCAGGC

KF620355

CGTGACGAGCTCGAGTCGGGGCGGGGAATCCAGCATTCTCTCGCCCCGCCTTGCATCCGTGGACCCATCGCTCGCGTTCCTGCGGCGACGGGTCCTACGGATCCTAACGAACCCCCGGCGCAGGTCGCGTCAAGGAGTACATGTGCGAACTTGGGCGGGTGCACGGCCGCGCGTCCCCGCGCGGCCCGTGCGGTTCCGCGCCCCTTCCACGCAGCGAACTGTATGACTCTCGGCAATGGATATCTAGGCTCTCGCATCGATGAAGAACGTAGCGAAATGCGATACTTGGTGTGAATTGCAGAATCCCGTGAACCATCGAGTCTTTGAACGCAAGTTGCGCCCGGAGCCGTCAGGCCGAGGGCACGTCTGCCTGGGCGACCAATAGGCGATCGCTCCCCTCCCCCCACCCCGAATGCGGTGGACTCGGGTGGTGGAAGCGGAAGCTGGCCATCCGTGAGCTTATAGGCATCGCACGATCCTTCGCGGTTGGCTCAATTCTCTCGACGATGTCTTGCCTCTGACGCATCACGTCGCGCGGTGGATCGCACTCGTTGCTTCACGTGCCGTGTCGTCCCGGCGAGACCTCCGTGGCCATTCCCAAAGCTGAGTATCCCCAGGTCAGGC
