# Supplementary figures and images for: Genetic species identification and population structure of Halophila (Hydrocharitaceae) from the Western Pacific to the Eastern Indian Ocean
Source: BMC Evol Biol. 2014 Apr 30;14:92. doi: 10.1186/1471-2148-14-92 (PMC4026155; doi:10.1186/1471-2148-14-92)

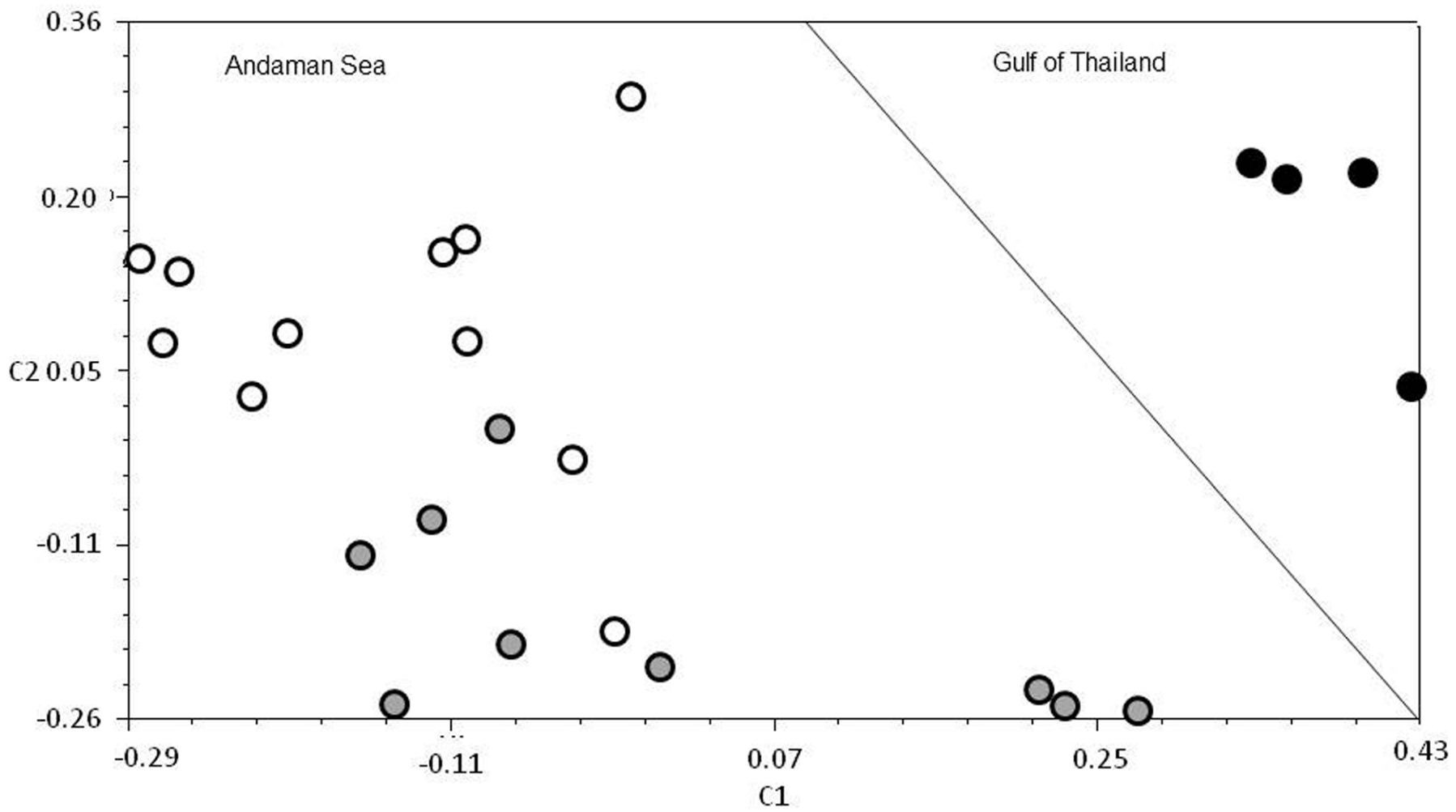

Supplement: Additional file 2 — Principal Coordinate Analysis (PCoA) based on 208 AFLP markers. There are two groups including the Gulf of Thailand and the Andaman Sea. Clustering of TH-tr and TH-sa is not significant. Abbreviations as in Figure 1. Symbols as in Figure 4. The matrix plot is processed by NTSYSpc, 2.20 [81]. [file 1471-2148-14-92-S2.pdf]

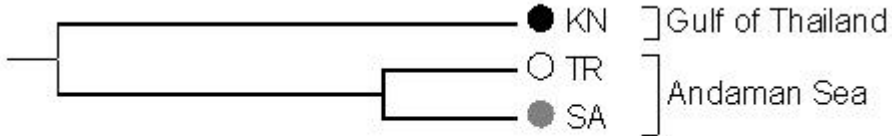

Nei's genetic distance (1978)

6

4

2

0

Supplement: Additional file 3 — Dendrogram of genetic distances among three populations of H. ovalis . Branch lengths were calculated by Nei [87]. Abbreviations as in Figure 1. Symbols as in Figure 4. Dendrogram was assessed by POPGENE 3.2 [82], edited by MEGA5.2 [46]. [file 1471-2148-14-92-S3.pdf]
